# PARP1-SNAI2 transcription axis drives resistance to PARP inhibitor, Talazoparib

**DOI:** 10.1038/s41598-022-16623-3

**Published:** 2022-07-21

**Authors:** Xia Ding, Zhou Zhu, John Lapek, Elizabeth A. McMillan, Alexander Zhang, Chi-Yeh Chung, Sara Dubbury, Jennifer Lapira, Sarah Firdaus, Xiaolin Kang, Jingjin Gao, Jon Oyer, John Chionis, Robert A. Rollins, Lianjie Li, Sherry Niessen, Shubha Bagrodia, Lianglin Zhang, Todd VanArsdale

**Affiliations:** 1 Oncology Research Unit, Pfizer, Inc., San Diego, CA 92121 USA; 2 Present Address: AstraZeneca, Inc., Gaithersburg, MD 20878 USA; 3 Present Address: Belharra Therapeutics, Inc., San Diego, CA 92121 USA; 4 Present Address: Odyssey Therapeutics., San Diego, CA 92121 USA; 5 Present Address: Bristol Myers Squibb., San Diego, CA 92121 USA; 6 Present Address: Turning Point Therapeutics., San Diego, CA 92121 USA; 7 Present Address: Genesis Therapeutics., San Diego, CA 92121 USA; 8 Present Address: Regeneron Pharmaceuticals, Inc., Tarrytown, NY 10591 USA

**Keywords:** Cancer, Cell biology

## Abstract

The synthetic lethal association between BRCA deficiency and poly (ADP-ribose) polymerase (PARP) inhibition supports PARP inhibitor (PARPi) clinical efficacy in *BRCA*-mutated tumors. PARPis also demonstrate activity in non-*BRCA* mutated tumors presumably through induction of PARP1-DNA trapping. Despite pronounced clinical response, therapeutic resistance to PARPis inevitably develops. An abundance of knowledge has been built around resistance mechanisms in *BRCA*-mutated tumors, however, parallel understanding in non-*BRCA* mutated settings remains insufficient. In this study, we find a strong correlation between the epithelial-mesenchymal transition (EMT) signature and resistance to a clinical PARPi, Talazoparib, in non-*BRCA* mutated tumor cells. Genetic profiling demonstrates that SNAI2, a master EMT transcription factor, is transcriptionally induced by Talazoparib treatment or PARP1 depletion and this induction is partially responsible for the emerging resistance. Mechanistically, we find that the PARP1 protein directly binds to *SNAI2* gene promoter and suppresses its transcription. Talazoparib treatment or PARP1 depletion lifts PARP1-mediated suppression and increases chromatin accessibility around *SNAI2* promoters, thus driving *SNAI2* transcription and drug resistance. We also find that depletion of the chromatin remodeler CHD1L suppresses SNAI2 expression and reverts acquired resistance to Talazoparib. The PARP1/CHD1L/SNAI2 transcription axis might be therapeutically targeted to re-sensitize Talazoparib in non-*BRCA* mutated tumors.

## Introduction

The selective sensitivity of *BRCA*-mutated cells over non-*BRCA* mutated cells to PARP inhibition has paved way for the success of PARP inhibitors in treating *BRCA*-mutated tumors with relatively low toxicity^1,2^. Currently, four PARPis including Olaparib, Niraparib, Rucaparib and Talazoparib have been approved for the treatment of *BRCA*-mutated tumors^3^. The synthetic lethal mechanism brought on by PARP inhibition and BRCA deficiency explains the specific vulnerability to PARPi in BRCA-mutated cells, particularly due to the homology-directed DNA repair (HDR) defect in these cells^4^. In addition to *BRCA*-mutated tumors, PARPis also demonstrate clinical activity in non-*BRCA* mutated tumors^5^. It is hypothesized that the underlying mechanism is associated with PARPi-induced PARP1 protein trapping on DNA^6^. More specifically, upon PARPi treatment, PARP1 auto-PARylation is inhibited and it can not be released from the DNA so that the PARP1-DNA trapping complex poses a barrier to impede essential cellular processes such as DNA replication, thus causing replication fork collapse and eventually cell death^7^.

Mechanisms of PARPi resistance in clinics largely include recovery of HDR ability by restoration of BRCA expression^8^. Other resistance mechanisms include enhanced DNA end resection (e.g. loss of the 53BP1-RIF1-REV7-Shieldin axis, the CST complex, HELB or DYNLL1), protection of replication fork stability (e.g. loss of PTIP, SLFN11, SMARCAL1 or EZH2), restoration of PARP1 signaling (e.g. loss of PARG or PARP1) and upregulation of P-glycoprotein^9^. Reduced 5-hydroxymethylcytosine (5hmc) content on the replication fork has also been reported to contribute to PARPi resistance^10^.

EMT is a critical cell state switching process in which epithelial cells lose polarity and cell–cell adhesion to become mesenchymal cells with enhanced differentiation (stemness) capability^11^. EMT is crucial for numerous physiological processes such as embryonic development and wound healing. In tumor biology, EMT program can be aberrantly activated and harnessed by tumor cells to acquire mesenchymal properties, which will allow tumor cells to gain aggressive phenotypes such as invasion, metastasis and drug resistance^12^. EMT is known to contribute to therapeutic resistance to a variety of chemotherapy drugs and targeted therapy drugs. For example, EMT drives cisplatin and doxorubicin resistance in multiple tumor models and through various mechanisms^13,14^. Activation of EMT has also been reported in epidermal growth factor receptor (EGFR)-mutant non-small cell lung cancer (NSCLC) models with acquired resistance to EGFR-targeted therapy beyond EGFR secondary alterations^15–17^. EMT has also been reported to be engaged in Olaparib resistance in *Brca2*-mutant mouse mammary tumor model^18^. However, the mechanism underlying Olaparib-induced EMT has not been elucidated.

Regulation of the phenotypic plasticity of EMT occurs at epigenetic level without alteration at genomic level. It is under the control of a sophisticated intracellular signaling generally regarded to be initiated by the TGFβ/SMAD pathway, followed by the downstream transcription regulatory network with the engagement of several major EMT-inducing transcription factors (EMT-TFs) including SNAI1, SNAI2, ZEB1/2 and TWIST1/2. EMT-TFs down-regulate the epithelial phenotype and up-regulate the mesenchymal phenotype, largely by suppressing E-Cadherin and cytokeratin expression and by increasing N-cadherin, vimentin and fibronectin expression^19^.

As a central EMT-TF and homolog to SNAI1 in the SNAIL transcription factor family, SNAI2 has been documented to play an important role in overcoming apoptosis and mediating drug resistance in tumor cells. SNAI2 has been suggested to be a pro-survival and prognostic factor in gastrointestinal stromal tumor^20^. In hematopoietic progenitors, it antagonizes p53-mediated apoptosis by repressing the pro-apoptotic gene *Puma*^14^. Multiple NSCLC cell lines also acquire cisplatin resistance through this mechanism^21^. SNAI2 also contributes to cisplatin resistance in ovarian cancer^22^. In breast cancer cells, SNAI2 upregulation is associated with an aggressive fulvestrant-resistant phenotype^23^. In patient derived xenograft (PDX) models from small cell lung cancer (SCLC) patients receiving Olaparib and Temozolomide combination treatment, high SNAI2 expression was found to have robust correlation with resistance to treatment^24^. The expression of SNAI2 can be regulated at both transcriptional level and post-translational level^25^. At transcription level, several pathways and transcription factors are known to either positively or negatively regulate SNAI2 transcription, including the TGFβ/SMAD pathway^26^, c-Myb^27^, p53^14^, KLF4 and FOXA1^28^, SOX3^29^, Per-Arnt-Sim transcription factor Singleminded-2 (SIM2)^30^, and ELF5, an ETS (E-twenty-six)-domain transcription factor^31^. *SNAI1* has also been reported to inhibit *SNAI2* via HDAC1^32^. The chromodomain helicase/ATPase DNA binding protein 1-like (CHD1L) chromatin remodeler protein has been recently shown to upregulate SNAI2 transcription^33^. SNAI2 expression level can also be regulated at post-transcriptional and post-translational level, for example, through miRNA and ubiquitin-dependent proteasomal degradation^25^.

Apart from the aforementioned bona fide EMT-TFs, PARP1 also plays important role in EMT. Specifically, PARP1 regulates EMT through transcriptional regulation of SNAIL, ZEB1 and HOXB7^34^. PARP1 also modifies TGFβ/SMAD pathway by PARylation of SMAD4^35^.

In the current study, we hypothesize that EMT contributes to PARPi resistance in non-*BRCA* mutated tumor cells and PARP1 serves as a transcriptional regulator of SNAI2. PARP1 chromatin occupancy suppresses *SNAI2* transcription, PARP inhibition by Talazoparib or PARP1 knockdown reduces chromatin-bound PARP1 protein on the *SNAI2* promoter to release the transcription suppression and subsequently induces EMT and drug resistance. Interestingly, CHD1L depletion can revert the induction of SNAI2 in cells with acquired resistance to Talazoparib and thus the acquired drug resistance. Targeting the PARP1/CHD1L/SNAI2 network might overcome therapeutic resistance to PARPis in treating non-*BRCA* mutated tumors.

## Result

### EMT signature correlates with intrinsic and acquired resistance to Talazoparib

**Figure 1:**
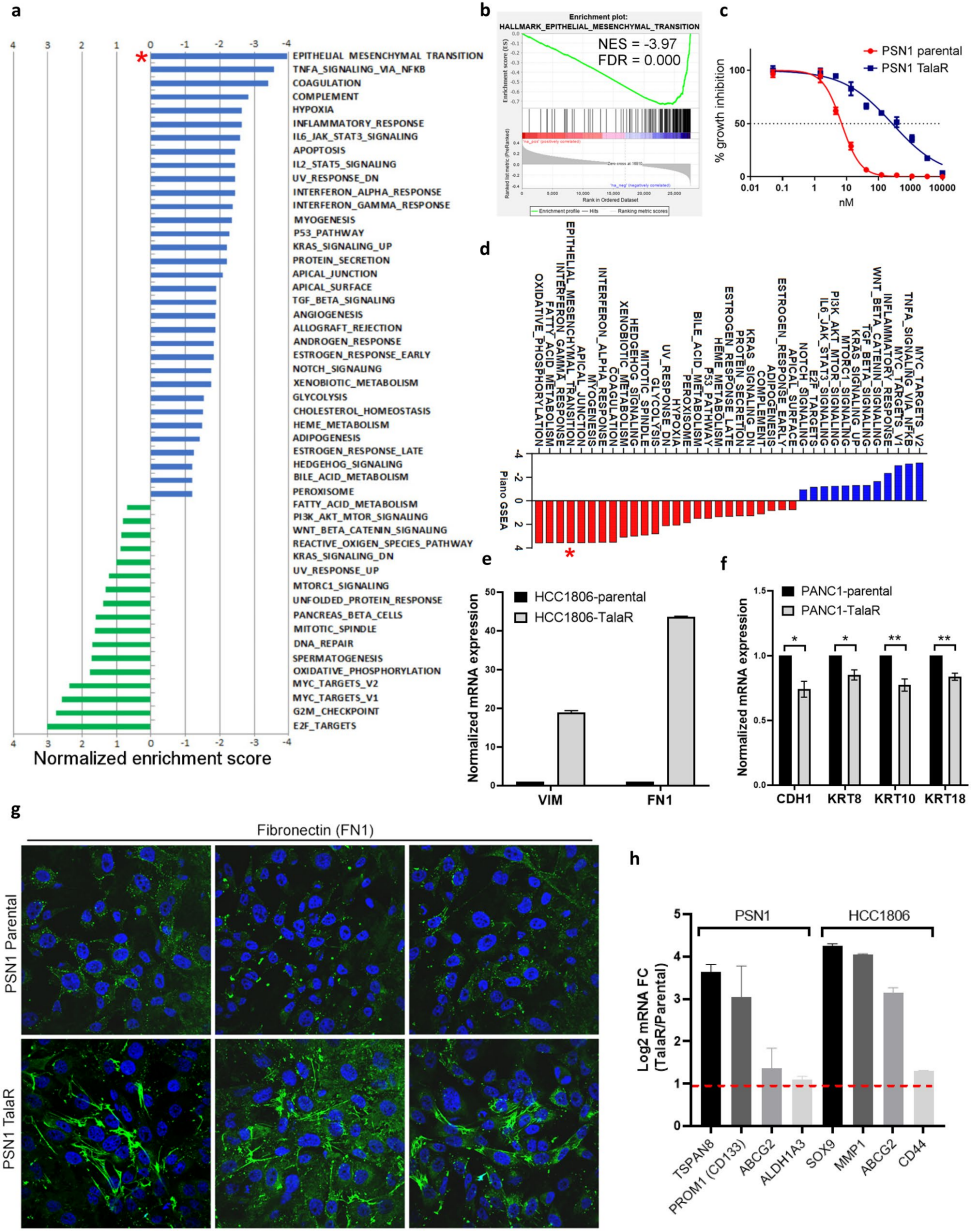
EMT signature correlates with intrinsic and acquired resistance to Talazoparib. (**a**) Pathway enrichment analysis showing pathway association with Talazoparib BOE. Negative normalized enrichment score indicates resistance, positive score indicates sensitivity. (**b**) Gene set enrichment analysis indicates correlation between EMT hallmark with intrinsic resistance to Talazoparib from BOE. (**c**) Talazoparib IC_50_ curves of parental PSN1 cells and cells with acquired resistance to Talazoparib. (**d**) Pathway enrichment analysis on total proteome of PSN1 parental and TalaR cells (n = 15). (**e**, **f**) qPCR showing mRNA level changes of mesenchymal and epithelial markers TalaR cells. (**g**) Immunostaining of fibronectin in PSN1 parental and TalaR cells. Three different fields in each group are shown. (**h**) qPCR showing upregulated cancer stem cell markers in PSN1 and HCC1806 TalaR cells vs parental cells. Dashed red line indicates twofold increase.

To study in vitro anti-tumor activity of Talazoparib in non-*BRCA* mutated tumors, we conducted Breadth of Efficacy (BOE) study by using CyQuant proliferation assay after 7-day Talazoparib treatment. We analyzed 334 *BRCA* wild type human tumor cell lines, which covered a broad spectrum of tumor lineages (Fig. S1a), including NSCLC, ovarian cancer, pancreatic ductal adenocarcinoma (PDAC), SCLC, breast cancer, bladder cancer, prostate cancer, hepatocellular carcinoma (HCC), gastric cancer, colorectal carcinoma (CRC), esophageal squamous cell carcinoma (ESCC), renal cell carcinoma (RCC), thyroid cancer, glioblastoma multiforme (GBM), head and neck squamous cell carcinoma (HNSCC), melanoma, cervical cancer and mesothelioma. Talazoparib exhibited a wide range of sensitivity across cell lines (Fig. S1b and Supplementary Table 1). We leveraged these data to identify potential determinants and biomarkers underlying such differential sensitivity in non-BRCA mutated tumor cell lines. Performing gene set enrichment analysis (GSEA), we identified pathways preferentially correlated with cellular Talazoparib sensitivity and resistance (Fig. 1a). Pathways enriched in sensitive cell lines included: “E2F targets”, “G2M checkpoint”, “MYC targets”, “Mitotic spindle” and “DNA repair” (Fig. 1a), highlighting a strong connection to the cell cycle and homology-directed repair (HDR) defect with PARPi sensitivity. High expression of PARP1 was found to be among genes with the strongest correlation with Talazoparib sensitivity (Fig. S1c), which supports the concept that PARP1-DNA trapping mediates PARPi cytotoxicity. Additionally, the top 10 highly correlates genes include: DHX9, PARP1, BUB1B, FAM189B, ANP32E, TAF5, ILF3, HNRNPU, DARS2 and RBMX. High expression of these genes correlates with increased Talazoparib sensitivity. Gene alteration correlation analysis between frequently mutated DDR genes^36^ and Talazoparib sensitivity showed that *ATR* and *NBN* mutations are significantly correlated with Talazoparib sensitivity (Fig S1d–f), demonstrating that other DDR gene mutations beyond *BRCA* mutations could provide Talazoparib sensitization. Pathways enriched in resistant lines highlighted the epithelial mesenchymal transition (EMT) pathway, which showed the most significant enrichment from our unbiased search (Fig. 1a,b), suggesting that it may play an important role in mediating intrinsic resistance to Talazoparib.

To further explore the correlation between EMT signature and Talazoparib resistance, we generated and profiled cell lines with acquired resistance to Talazoparib. Talazoparib-sensitive non-*BRCA* mutated cell lines were subjected to gradually increasing concentration of Talazoparib (from 10 to 100 nM) to allow for development of acquired resistance. These cell lines included PSN1, PANC1, SW1990 and HCC1806 cells, all with the IC_50_ values under 30 nM in parental models. Cells with acquired resistance to Talazoparib (designated as TalaR) were developed after 3–4 months selection in drug media (Fig. 1c and Fig. S2a). TalaR cells were cross-resistant to other PARPis (Fig. S2b), suggesting this resistant phenotype was not specific to Talazoparib. Characterization of these resistant lines that were cultured in drug media demonstrated clearly reduced PAR levels (Fig. S2c), and PARP1-DNA trapping levels (induced with MMS) that were comparable to the parental cells (Fig. S2d). Moreover, TalaR cells (maintained in drug media) harbored more DNA damage as indicated by γH2AX foci (Fig. S2e,f). These collective results suggest that in TalaR cells, Talazoparib was still fully engaging its cellular target PARP1, thus the resistance mechanism is unlikely through the reactivation of PARP1. We found TalaR cells were cross-resistant to cisplatin (Fig. S2g) suggesting possible trapping-independent resistance mechanism. We also showed that while TalaR-M cells (TalaR-M is TalaR cells maintained in drug media, M stands for maintained) had ablated PAR level, TalaR-DF cells (TalaR-DF is TalaR cells cultured in drug free media for 4-weeks, DF stands for drug free;) had nearly comparable PAR level to parental cells, indicating PARP1 protein in TalaR cells is still carrying out its bona fide function in PARylation and repairing DNA (Fig. S2h).

To illuminate resistance mechanism in TalaR cells, we performed whole proteome sequencing by mass spectrometry (MS) and transcriptome analysis by RNA sequencing (RNAseq). Proteome and transcriptome profiling highlighted concordant up-regulation of the EMT pathway in TalaR cells (PSN1, HCC1806, PANC1) (Fig. 1d and Fig. S2i–k). We validated EMT hallmarks including increased mRNA expression of vimentin (VIM) and fibronectin (FN1), increased immunostaining signal of FN1 and decreased mRNA expression of E-cadherin (CDH1), cytokeratin 8 (KRT8), cytokeratin 10 (KRT10) and cytokeratin 18 (KRT18) in TalaR cells (Fig. 1e–g). Tumor cells undergoing EMT acquire cancer stem cell (CSC) properties, which are manifested by more aggressive phenotypes such as migration and invasion^12^. The transcriptomic profiling indicated PSN1 and HCC1806 TalaR cells also had increased “stemness” as shown by up-regulated CD133, ABCG2, TSPAN8, ALDH1A3, SOX9, MMP1, and CD44 (Fig. 1h). Collectively, these results suggest EMT signature is associated with both intrinsic and acquire resistance to Talazoparib.

### Cell line-dependent reversibility of acquired Talazoparib resistance

**Figure 2:**
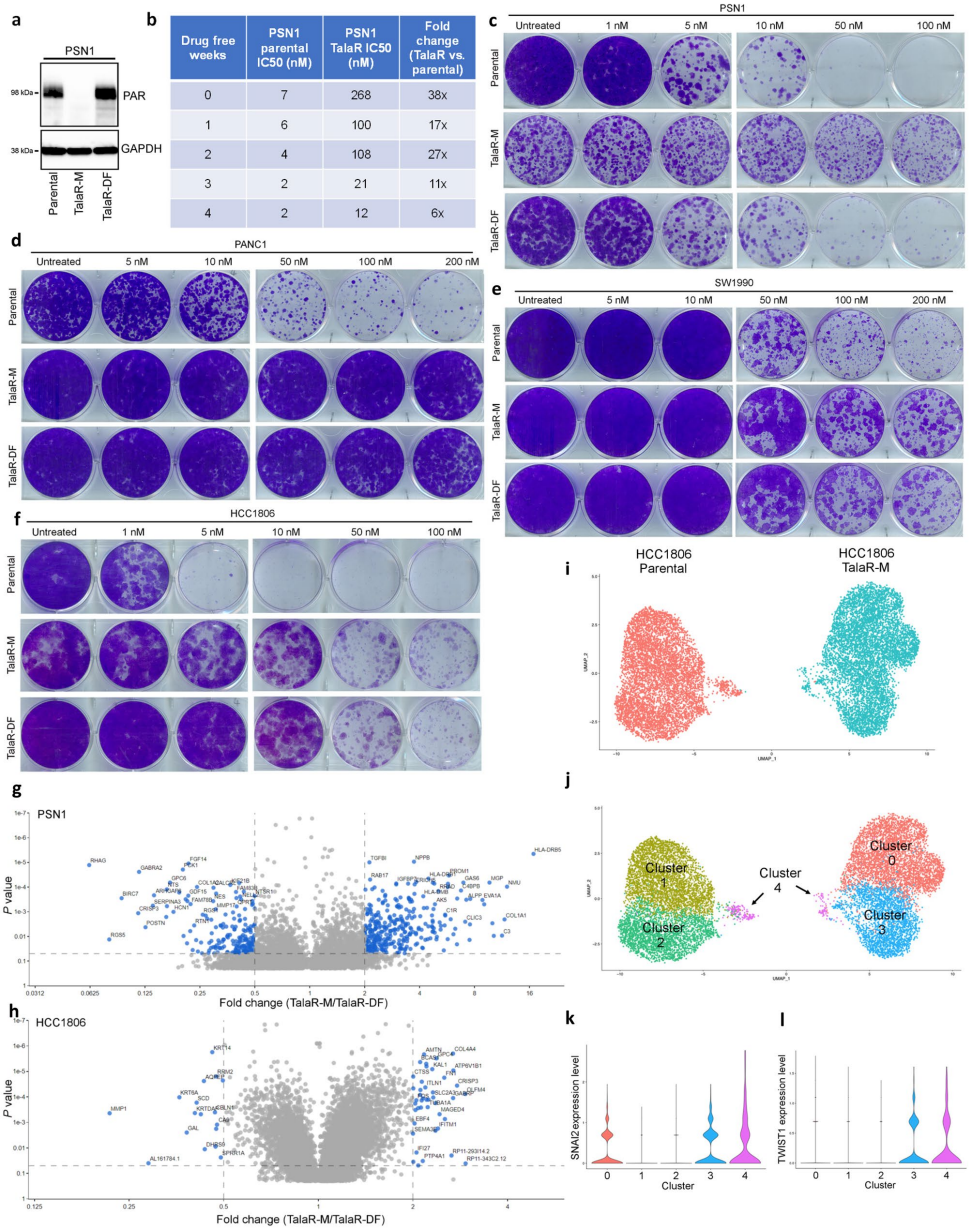
Cell line-dependent reversibility of acquired Talazoparib resistance. (**a**) Immunoblot showing PAR level of PSN1 parental cells, TalaR cells maintained in media with Talazoparib (TalaR-M) and TalaR cells cultured in drug-free media for 4 weeks (TalaR-DF). (**b**) Talazoparib IC_50_ chart showing reversibility of acquired resistance in PSN1 cells over 4 weeks after drug withdrawal. (**c**–**f**) Clonogenic assay showing Talazoparib sensitivity of parental and acquired resistant cells in PSN1, PANC1, SW1990 and HCC1806 cells which were maintained in drug media (Tala-M) and in drug free media for 4 weeks (Tala-DF). (**g**, **h**) Volcano plots showing differentially expressed genes between TalaR-M and TalaR-DF in PSN1 and HCC1806 cells. (**i**, **j**) UMAP representations of HCC1806 parental and resistant subpools, colored by cell line (**i**) or cluster identity (**j**). (**k**, **l**) Violin plots of SNAI2 (**k**) and TWIST1 (**l**) gene expression profile across subpopulation clusters shown in (**j**).

To understand mechanism of acquired resistance, we examined whether the acquired resistance was reversible. After drug withdrawal, PSN1 TalaR cells started to gradually become re-sensitized and after 4 weeks of drug withdrawal, they became almost equally sensitive to Talazoparib as parental cells. In addition, their PAR levels were also restored to the levels observed in parental cells (Fig. 2a–c). Meanwhile, PANC1, SW1990 and HCC1806 TalaR cells showed irreversible resistance upon drug withdrawal (Fig. 2d–f). To further examine the phenotypic difference, we compared the transcriptome of TalaR cells maintained in drug media (Tala-M, M stands for maintained) over TalaR cells in drug free media for 4-weeks (TalaR-DF, DF stands for drug free). The data showed that there were a large number of differentially expressed (DE) genes between TalaR-M and TalaR-DF in PSN1 cells, whereas the difference between TalaR-M and TalaR-DF in HCC1806 cells was far less in comparison (Fig. 2g,h), indicating cell line-dependent reversibility of acquired Talazoparib resistance.

To characterize the origin of resistance in HCC1806, we performed single cell RNA sequencing (scRNAseq) comparing the HCC1806 parental and TalaR cell populations. We sequenced 6594 cells of HCC1806 parental or 6944 cells of HCC1806 TalaR followed by clustering cells based on similarity of gene expression across all populations. This analysis indicated that HCC1806 parental cells were made up of 3 subpopulations Clusters 1, 2, and 4 (Fig. 2i,j) with a majority (~ 97.4%) of cells residing in clusters 1 and 2. Conversely, HCC1806 TalaR cells were comprised of 3 subpopulations Clusters 0, 3, and 4, with a majority (~ 98.8%) of cells residing in two clusters completely independent from the parental population (Clusters 0 and 3) (Fig. 2i,j). Interestingly, the Cluster 4 sub-population was observed at low frequencies in both the parental and resistant HCC1806 populations (~ 2.5% and ~ 1.2% respectively). Gene expression analysis of cluster 4 indicated that this subpopulation was dominated by markers of “stemness”, including up-regulated SOX4, IGF1, IGF2, TNF, FOXO3, KLF4, as well as several stress response pathways to maintain cellular homeostasis, such as ATF3, ATF4, DDIT3 and DDIT4^37–42^. Importantly, EMT-TF expression, including SNAI2 and TWIST1, was upregulated in cluster 4 (Fig. 2k,l). These data suggest that there might be a small, pre-existing resistant population in parental cells enriched in high EMT-TF expression that reconstitutes the HCC1806 TalaR population under Talazoparib selection.

Collectively, these results suggest that the reversibility of acquired resistance to Talazoparib differs in an idiosyncratic way across different cell lines.

### Talazoparib induces EMT signature and SNAI2 expression which partially drives resistance

**Figure 3:**
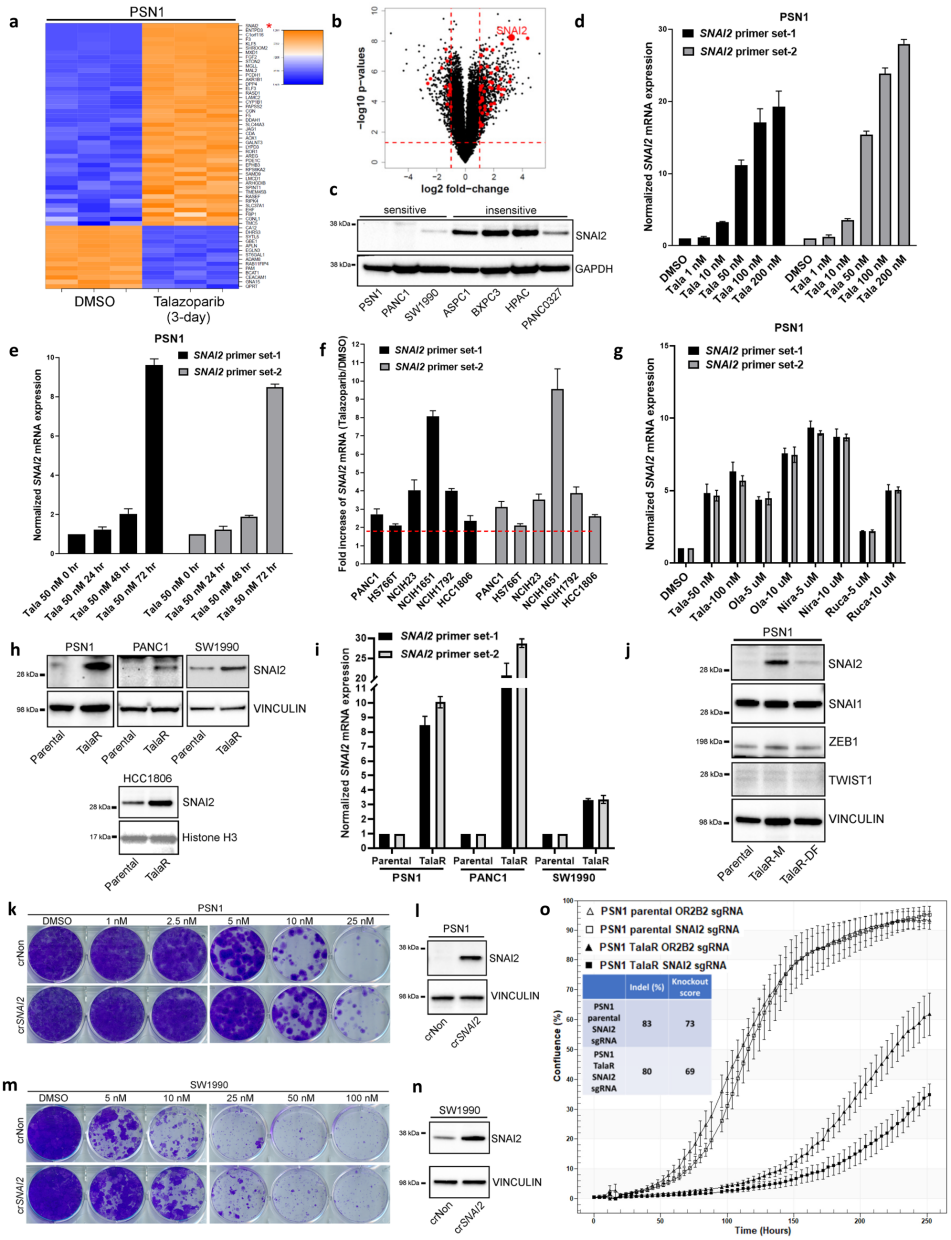
Talazoparib induces EMT signature and *SNAI2* expression which partially drives resistance. (**a**, **b**) Heatmap and volcano plot of RNAseq showing top differentially expressed EMT genes under 3-day Talazoparib treatment in PSN1 cells. (**c**) Immunoblot showing SNAI2 expression in Talazoparib sensitive and insensitive PDAC cell lines. (**d**, **e**) qPCR showing dose- and time-dependent induction of SNAI2 under Talazoparib treatment in PSN1 cells. Two independent sets of *SNAI2* primers were used. (**f**) qPCR showing Talazoparib at 100 nM induces *SNAI2* expression across different cell lines. Two-fold increase was highlighted in red dashed line. (**g**) qPCR showing induction of SNAI2 by different clinical PARPis. (**h**) Immunoblot showing SNAI2 upregulation in cell lines with acquired resistance to Talazoparib. (**i**) qPCR showing *SNAI2* mRNA level in parental and TalaR cells. (**j**) Immunoblot showing expression of SNAI2 and other EMT-TF in PSN1 parental, TalaR-M and TalaR-DF cells. (**k**, **m**) Clonogenic assay showing cells gained resistance to Talazoparib after SNAI2 by CRISPRa in PSN1 and SW1990 cells. (**l**, **n**) Immunoblot showing induced SNAI2 expression by CRISPRa in PSN1 and SW1990 cells. (**o**) IncuCyte analysis curves showing growth of PSN1 parental and TalaR cells after SNAI2 knockout. Insert shows Indel% and knockout score.

To further elucidate how the drug-induced plasticity contributes to acquired resistance to Talazoparib, we examined whether EMT activation is an adaptive response to Talazoaprib that allows the cells to develop drug resistance. We conducted RNAseq on a panel of PDAC cell lines treated with Talazoparib for 72 h. Transcriptomic changes highlighted induction of EMT signature across different cell lines (Fig. S3a–c). In PSN1 cells, one of the top up-regulated EMT genes was SNAI2 (Fig. 3a,b), a critical transcription factor contributing to EMT. High SNAI2 expression was also correlated with Talazoparib intrinsic resistance in PDAC cell lines (Fig. 3c). Because of its critical role as a master EMT-TF, we speculated SNAI2 induction might be crucial for the development of acquired resistance to Talazoparib. We validated by qPCR that Talazoparib-induced SNAI2 expression was both dose- and time-dependent (Fig. 3d,e). Meanwhile, TWIST1 and SNAI1 expression was not upregulated by Talazoparib treatment (Fig. S3d). Besides PSN1 cells, there was a universal increase of *SNAI2* mRNA expression across other PDAC, NSCLC and breast cancer cell lines in response to 72 h Talazoparib treatment (Fig. 3f). To study Talazoparib-induced SNAI2 in the context of BRCA status, we depleted BRCA1 in PSN1 (PSN1 is BRCA1/2-proficient) and observed increased Talazoparib sensitivity (Fig. S3e,f). We were also able to observe induction of SNAI2 in BRCA1-mutated ovarian and breast cancer cell lines COV362 and MDAMB436 (Fig. S3g,h). Despite the varying fold increase, this result suggests that Talazoparib-induced SNAI2 expression could be a rather general phenomenon regardless of BRCA1/2 status. Furthermore, the induction of SNAI2 was not restricted to Talazoparib, as other clinical PARPis, including Olaparib, Niraparib and Rucaparib, also induced SNAI2 expression within 72 h treatment (Fig. 3g).

Next, we examined whether there was also a *SNAI2* increase in TalaR cells developed from longer-term Talazoparib selection. Consistently, there was a pronounced SNAI2 upregulation at both protein and mRNA levels in TalaR cells developed from PSN1, PANC1, SW1990 and HCC1806 cells as compared to parental cells (Fig. 3h,i). Interestingly, in PSN1 cells where resistance is reversible, increased SNAI2 expression was also reversible with the drug withdrawal (Fig. 3j). These results suggest the drug-induced plasticity manifested by *SNAI2* increase might provide survival advantage and thus be naturally selected under drug pressure.

To explore the causality between SNAI2 and Talazoparib resistance, we performed a gain-of-function experiment by using CRISPR activation (CRISPRa) to stably enhance the expression of endogenous SNAI2 in PSN1 and SW1990 cells. After SNAI2 was turned up by CRISPRa, both cell lines gained resistance to Talazoparib as shown by increased clonogenicity (Fig. 3k–n). We were also able to observe increased resistance to Talazoparib in PSN1 cells with transiently overexpressed SNAI2 by using CyQuant cell proliferation assay (Fig. S4a). Consistently, we found that SNAI2 overexpression not only induced fibronectin (FN1) expression similar to Talazoparib did (Fig. S4b,c), but also conferred resistance to Talazoparib-induced apoptosis (Fig. S4d). In light of SNAI2’s function in EMT and anti-apoptosis, these results suggest SNAI2 overexpression might confer Talazoparib resistance through both mechanisms. Next, we performed loss-of-function experiment in which SNAI2 expression was stably knocked down in PSN1 cells, we observed a concordant sensitization effect to Talazoparib (Fig. S4e,f). Furthermore, we tested whether the growth of TalaR in Talazoparib-containing media was partially dependent on SNAI2, we generated a knockout of SNAI2 by CRISPR/Cas9 in TalaR cells and monitored cell growth by IncuCyte. We were able to observe a slower growth in PSN1 TalaR cells (maintained in Talazoparib-containing media) when SNAI2 was depleted, while SNAI2 knockout to similar level in PSN1 parental cells did not affect growth (Fig. 3o). Collectively, these results suggest that SNAI2 induction is not only correlated with resistance, but is also one of the drivers of acquired resistance to Talazoparib.

### PARP1 directly regulates *SNAI2* expression through transcription regulation

**Figure 4:**
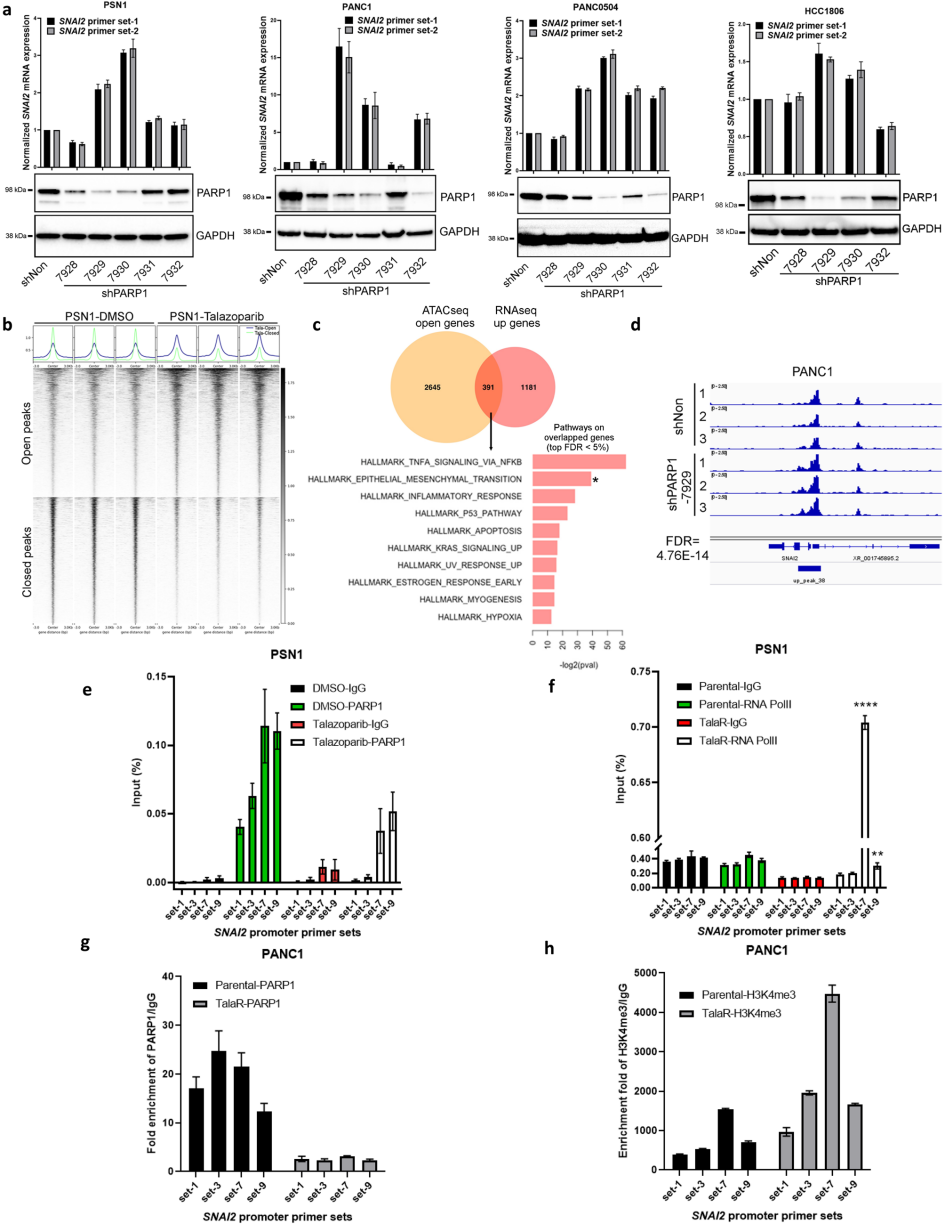
PARP1 protein directly regulates SNAI2 transcription through transcription suppression. (**a**) qPCR and immunoblot showing KD level and SNAI2 expression in cell lines with PARP1 stable KD. (**b**) Summary of open and closed peaks in ATACseq on PSN1 cells treated with DMSO or Talazoparib for 72 h. Three biological replicates are shown for each group. (**c**) Venn diagram showing the number of overlapped gene between ATACseq open genes and RNAseq up genes. Waterfall plots shows enriched pathways from overlapped genes. (**d**) Representative ATACseq peaks showing chromatin accessibility changes on SNAI2 promoter in PANC1 cells with PARP1 stable KD. Three biological replicates are shown for each group. (**e**) ChIP-qPCR by using IgG or PARP1 antibody pulldown in PSN1 cells treated with DMSO or Talazoparib (50 nM, 72 h). Four pairs of primes surrounding SNAI2 promoter (set-1, -3, -7 and -9) ranging from − 2 to + 0.7 kb TSS were used. Same sets of primers were used below. (**f**) ChIP-qPCR by using RNA polymerase II (RNA PolII) antibody pulldown in PSN1 parental and TalaR cells. (**g**, **h**) ChIP-qPCR by using PARP1 antibody or H3K4me3 antibody pulldown in PANC1 parental and TalaR cells.

To probe the mechanism of Talazoparib induced *SNAI2* expression, we asked whether PARP1 knockdown (KD) could phenocopy Talazoparib-induced *SNAI2*. Stable KD of PARP1 in PSN1, PANC1, PANC0504 and HCC1806 cells resulted in a general increase of *SNAI2* expression (Fig. 4a) without an induction of *TWIST1 or SNAI1* (Fig. S5a,b). These results suggest that PARP1 might directly regulate and suppress *SNAI2* transcription.

To tease out the role of PARP1 from the many pathways that can regulate SNAI2 transcription, we first examined the TGFβ/SMAD pathway, because it is a well-established contributor to EMT and SNAI2 transcription^25^. PARP-1 has also been shown to attenuate SMAD-mediated transcription^35^. We examined whether there is any crosstalk between Talazoparib/PARP1 KD-induced *SNAI2* expression and TGFβ-induced *SNAI2* expression. TGFβ substantially induced SMAD2 phosphorylation on Serine 465 and Serine 467, but Talazoparib did not have any effect on SMAD2 phosphorylation in both SMAD4-deficient PSN1 and SMAD4-proficient PANC1 cell lines (Fig. S5c). Next, we studied whether inhibiting TGFβ pathway can revert Talazoparib-induced SNAI2 expression. LY3200882 is a potent and selective TGFβR1 inhibitor. LY3200882 potently blocked TGFβ-induced *SNAI2* expression in PSN1 cells, but it did not block Talazoparib-induced *SNAI2* expression (Fig. S5d). These results suggest that Talazoparib does not induce TGFβ pathway activation and Talazoparib-induced *SNAI2* expression is independent of TGFβ pathway activation. Activation of p53 could also contributes to *SNAI2* induction^14^. PARP inhibitors have been reported to induce and potentiate p53 activation^43,44^. To test the potential involvement of p53, we efficiently knocked down p53 by siRNA and observed that Talazoparib-induced *SNAI2* induction was not attenuated (Fig. S5e,f), thus we could exclude the possibility that SNAI2 induction was due to p53 activation.

The role of PARP1 as a transcription factor has been well documented, and PARP1 nucleosome occupancy regulates transcriptional outcome through various mechanisms^34,45^. To this end, we next asked whether there are chromatin accessibility changes associated with Talazoparib treatment or PARP1 KD that could possibly account for increased SNAI2 expression. We performed Assay for Transposase Accessible Chromatin-sequencing (ATACseq) by using PSN1 cells treated with Talazoparib. The TSS plot showed drastic changes in chromatin accessibility with both open and closed chromatin (Fig. 4b). By overlaying genes with open chromatin as manifested in ATACseq with up-regulated differential genes as manifested in RNAseq, we were able to identify 391 common genes, and hallmark pathway analysis of these common genes highlighted the EMT pathway (Fig. 4c), suggesting the possibility that Talazoparib-induced up-regulation of EMT genes is through increasing chromatin accessibility of these genes. Consistently, ATACseq performed by using PANC1 cells with PARP1 stable KD, which had pronounced increase in SNAI2 expression as shown in Fig. 4a, also showed increased peak of ATACseq track in *SNAI2* promoter region (Fig. 4d). This result provided direct evidence that PARP1 KD increases *SNAI2* chromatin accessibility which may lead to increased SNAI2 expression.

Next, we examined the direct binding of PARP1 on *SNAI2* promoter by by ChIP-qPCR. In PSN1 cells treated with Talazoparib for 72 h, we conducted ChIP by pulling down with PARP1 antibody followed by qPCR using four sets of primers (set-1, 3, 7 and 9) surrounding *SNAI2* promoter. These primers span 2 kb upstream of TSS and 0.7 kb downstream of TSS. As shown in Fig. 4e, we detected enrichment of PARP1 on *SNAI2* promoter at basal level (DMSO) by all four sets of primers. Importantly, the enrichment was attenuated under Talazoparib treatment. This finding suggests PARP1 directly binds to *SNAI2* promoter and Talazoparib treatment reduces the level of PARP1 on the promoter possibly to increase SNAI2 transcription. To strengthen this hypothesis, we conducted ChIP-qPCR in PSN1 parental and TalaR cells by pulling down with RNA polymerase II (RNA PolII) and detected RNA PolII binding by the same four sets of SNAI2 promoter primers. In parental cells, enrichment of RNA PolII on SNAI2 promoter was minimal. Strikingly, upon Talazoparib treatment, the enrichment detected by primer set-7, which is primer set spanning right at the TSS site, increased dramatically (Fig. 4f). There was also a slight increase in enrichment as detected by primer set-9, which is the set that is 0.7 kb downstream of TSS. This suggests that Talazoparib lifts the suppression of PARP1 on *SNAI2* promoter to allow RNA PolII-mediated gene transcription. To study whether the lift of transcription suppression is associated with histone marker, we also performed ChIP-qPCR by pulling down with an antibody against H3K4me3 which is a histone marker for active gene transcription. As in PSN1 cells, there was PARP1 enrichment on *SNAI2* promoter in PANC1 parental cells and this enrichment was reduced in TalaR cells (Fig. 4g). Importantly, we also found an increased H3K4me3 signal on *SNAI2* promoter, especially around the TSS region as detected by primer set-7 (Fig. 4h). This suggests that Talazoparib-induced SNAI2 transcription is associated with increased active histone marker. Combined with the ATACseq result, we hypothesize that the PARP1 protein regulates transcription of *SNAI2* gene by serving as a transcription suppressor that directly binds to *SNAI2* promoter. Talazoparib lifts the suppressive binding of PARP1 on *SNAI2* promoter to allow histone modification towards increased chromatin accessibility and active engagement of RNA PolII and eventually induces *SNAI2* transcription.

### CHD1L depletion suppresses *SNAI2* expression and re-sensitizes cells with acquired resistance to Talazoparib

**Figure 5:**
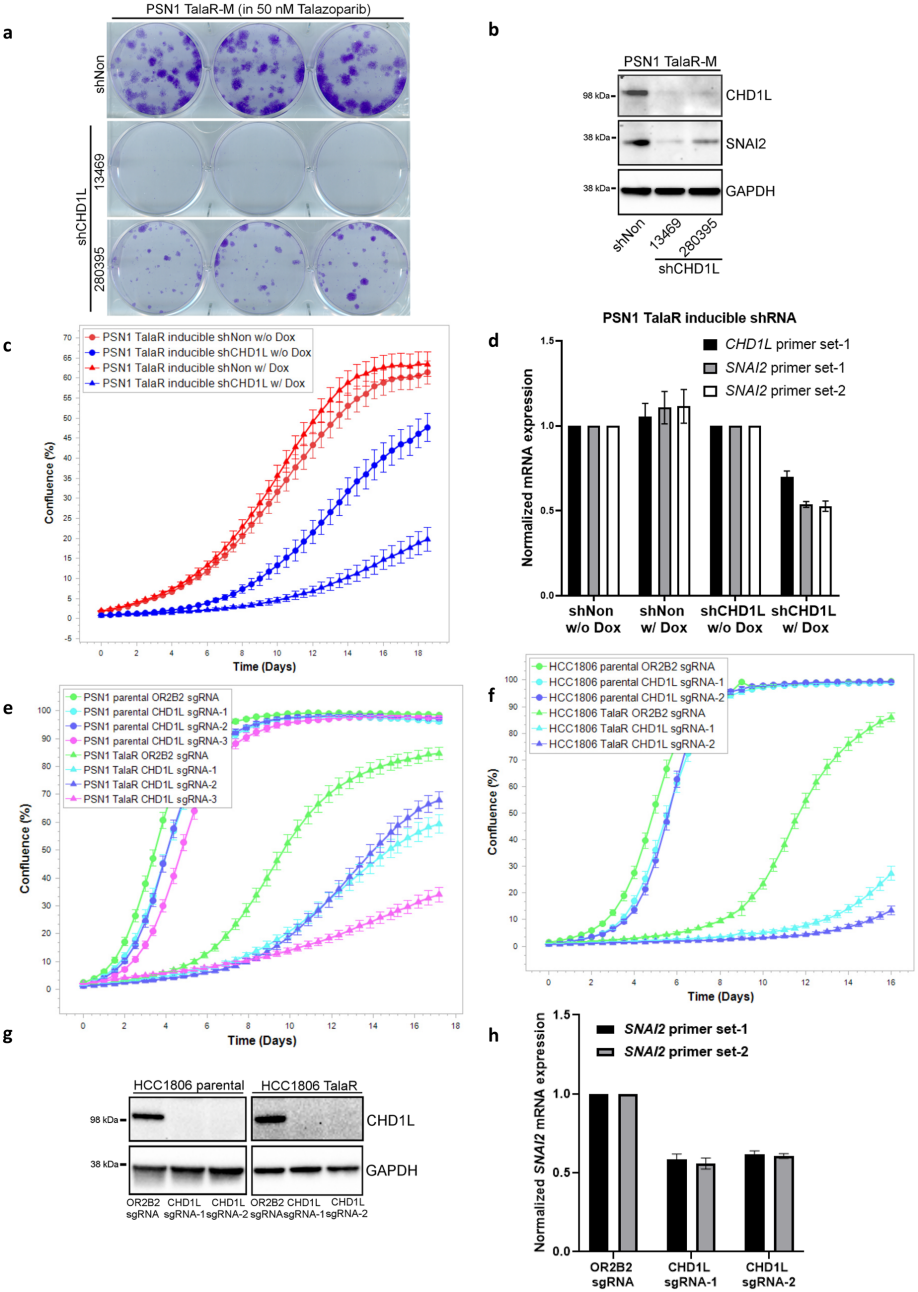
CHD1L depletion re-sensitizes cells with acquired resistance to Talazoparib. (**a**) Clonogenic assay showing growth of PSN1 TalaR-M cells with CHD1L stable knockdown. (**b**) Immunoblot showing expression level of CHD1L and SNAI2 in CHD1L stable knockdown cells as in (**a**). (**c**) IncuCyte curves showing the growth PSN1 TalaR-M with inducible CHD1L knockdown with and without doxycycline induction. (**d**) qPCR showing mRNA level of *CHD1L* and *SNAI2* in PSN1 TalaR-M with inducible CHD1L knockdown with and without doxycycline induction as in (**c**). (**e**) IncuCyte curves showing the growth of PSN1 parental and TalaR-M with CHD1L knockout. (**f**) IncuCyte curves showing the growth of HCC1806 parental and TalaR-M with CHD1L knockout. (**g**, **h**) Immunoblot and qPCR showing CHD1L and SNAI2 level in HCC1806 parental and TalaR-M with CHD1L knockout as in (**f**).

To find re-sensitization targets against TalaR cells, we compiled genome wide CRISPR screening data for PARPi (including Olaparib and Talazoparib) sensitization targets in the public domain. Since we found Talazoparib-induced adaptive changes in chromatin accessibility contributed to resistance, we focused on the sensitization targets that are categorized as chromatin remodelers. Across three independent studies using cell lines with various genetic background^46–48^, chromodomain helicase/ATPase DNA binding protein 1-like gene (*CHD1L*) repeatedly emerged as one of the top sensitization targets. CHD1L inhibition has recently been validated to sensitize to PARPi through various mechanisms, including inducing PARP1/2 trapping and BER deficiency^49–51^. CHD1L also contributes to stemness in pluripotent cells, it regulates pluripotency, differentiation and invasiveness of cancer cells; furthermore, inhibition of CHD1L was reported to down-regulate SNAI2 expression^33,52–54^. These studies prompted us to examine CHD1L as a potential target to revert Talazoparib resistance through the link to SNAI2 regulation. Following knock down of CHD1L in PSN1 TalaR cells by shRNA, we examined clonogenic ability. Two independent CHD1L shRNA efficiently down-regulated CHD1L, reduced SNAI2 expression and inhibited clonogenicity of TalaR cells grown in media containing 50 nM Talazoparib as in all experiments below (Fig. 5a,b). We further generated PSN1 TalaR cells with inducible shRNA against CHD1L and observed that with the doxycycline induction, proliferation of TalaR cells was slowed down as monitored by IncuCyte and there was a concordant reduction of both CHD1L and SNAI2 at mRNA level (Fig. 5c,d). Next, we compared CHD1L dependency between parental and TalaR in PSN1 and HCC1806 cells. We depleted CHD1L by CRISPR/Cas9 and monitored cell growth by IncuCyte. We did head-to-head comparison on the growth of parental cells (PSN1 and HCC1806) with the corresponding TalaR cells. Even though CHD1L depletion in both parental and TalaR cells were equally efficient, it had no significant effect on the growth of parental cells in both cell lines, but it remarkably reduced growth of TalaR cells in Talazoparib containing media (Fig. 5e–g). In the meantime, CHD1L depletion also reduced SNAI2 level in TalaR cells (Fig. 5h). Our data suggested that CHD1L might be targeted to revert the induced SNAI2 expression thus re-sensitize tumor cells that have acquired resistance to Talazoparib.

## Discussion

PARP inhibitors have been proven to be clinically successful in treating patients with both *BRCA*-mutated and non-*BRCA* mutated tumors. In the non-*BRCA* mutated setting, stratifying tumors to predict PARPi response and overcoming resistance remains to be fully understood. In our study, we found that EMT signature correlates with both intrinsic and acquired resistance to Talazoparib, which highlights EMT signature as both a predictive biomarker and a mechanistic driver for PARPi resistance. Based on our data, we propose Talazoparib lifts PARP1-mediated suppression of *SNAI2* transcription and drives resistance towards itself.

In our acquired resistance models in PSN1 and HCC1806 cells, we observed selection of pre-existing resistant population and drug-induced plasticity at epigenetic level both contribute to the development of acquired resistance to Talazoparib. We also noticed that cell lines with different genetic background show idiosyncrasy in employing these two models to develop resistance. Further studies are needed to uncover what underlies the choice of these different mechanisms in different cell lines. Literature suggests that intratumor heterogeneity (ITH) might play a role during this process^55^. In this study, tumors with higher ITH undergo selection of pre-existing population, as there is a relatively larger genetic pool that can be selected from by the drug pressure. Whereas tumors with lower ITH are more likely to undergo plastic transformation as the cell genetic diversity is low for certain population to be enriched by drug selection, thus cancer cells employ epigenetic changes to survive. In our study, it will be intriguing to explore whether there is a distinct ITH pattern in PSN1 (reversible resistance) and HCC1806 (irreversible resistance) cells that could possibly explain why the former is more plastic than the latter when it comes to developing resistance to Talazoparib.

The basic hypothesis arising from our scRNAseq data is that cluster 4 is a "stem cell like progenitor population" giving rise to the TalaR cells with high EMT markers, but this remains largely a speculation at this point as we do not have other evidence (e.g. pseudo-time analysis) to support the hypothesis. Nevertheless, cluster 4 is found amongst the parental and the resistant at a low frequency, and expressing the EMT transcription factors, so the idea would be that in the resistant lines those progenitors give rise to a line with high EMT TFs. The SNAI2 data lines up very well with this as we see it expressed in cluster 4 (the progenitors) and in the two resistant clusters (cluster 0 and cluster 3). The TWIST1 data is a little more confounding-we see it in the "progenitor population" as well as one of the two resistant clusters (cluster 3). One has to keep in mind that a major limitation of scRNAseq is the ability to detect lowly expressed genes (like TFs), this might be a capture issue but it remains a formal possibility that there are two distinct resistant clusters (0 and 3), one which expresses TWIST1 and one that does not.

We want to point out that although EMT is highlighted as a driver to Talazoparib resistance in our study, it is not the only contributing pathway, and the contribution of other pathways must not be underestimated. The fact that both our gain of function and loss of function experiments on SNAI2 only moderately alter Talazoparib sensitivity clearly suggests contribution of other pathways. Besides EMT signature, our proteomics and RNAseq data comparing TalaR and parental cells also highlighted pathways such as KRAS signaling, Hedgehog pathway and hypoxia pathway, that are known to play important roles in drug resistance^56–58^. These pathways, including EMT, are also highly intertwined, making it difficult to dissect which one is more dominant than the others. In addition, when considering the contribution of SNAI2 to Talazoparib resistance, the multifaceted cellular functions of SNAI2 beyond its role in EMT must be taken into account, as it is also involved in the anti-apoptotic and DNA repair pathways. These confounding factors epitomize the complexity of how tumor cells acquire resistance to Talazoparib.

The regulation of SNAI2 transcription is also controlled by a sophisticated network of factors, including a vast number of transcription factors that can regulate its expression^25^. In our study, we only chose to study and exclude two possibilities, the TGFβ and p53 pathways, because these two pathways are known to be related to PARP1 and PARP inhibition, although other transcriptional regulation might also lead to Talazoparib-induced SNAI2 upregulation. Pathways such as Notch, Wnt and IFN-γ pathways, which are upregulated in TalaR cells and have been reported to be associated with EMT and SNAI2, may also contribute to SNAI2 induction in our scenario^59–61^. The results of our studies support a role for PARP1 as a transcription suppressor of SNAI2. PARP1 activation of gene transcription has been well documented in various context has been shown to be dependent on PARylation activity. In our ATACseq and RNAseq results, we observed both activation and suppression of gene transcription when PARP is inhibited. The PARP1-mediated suppression could be due to the basal level of PARylation being required in order for PARP1 to bind to certain promoters and to occupy chromatin regions to suppress transcription. When PARylation is inhibited, PARP1 can no longer bind to these regions and corresponding genes may become activated. This process can be highly dependent on genomic landscape. How PARP inhibition promotes RNA polII binding also remains to be further explored: PARylated PARP1 might occupy promoter regions in a competitive manner against PolII to suppress PolII-mediated gene transcription. When PARylation is inhibited by Talazoparib, PARP1 can no longer bind to these regions, this allows PolII to take its place and mediate activation of corresponding genes. Another hypothesis could be that PARylation of epigenetic factors by PARP1 could reduce active histone marks to attenuate gene transcription, once PARylation of these epigenetic factors is inhibited by Talazoparib, active histone marks will resume to allow active gene transcription. One example is that epigenetic factor NSD2 (writer of H3K36me2, a histone mark for gene activation) has been shown to be PARylated by PARP1 and PARylated NSD2 loses its methyltransferase activity^62^. These hypotheses raise an intriguing mechanism that while PARPi is inhibiting PARP1 activity in order to inhibit cell proliferation, the loss of activity and reduced auto-PARylation backfires by driving acquired resistance against itself. It is also worth noting that PARPi induces PARP1-DNA trapping, which contradicts our results showing that in certain regions the chromatin accessibility is increased under PARPi treatment. We reason that trapping can be highly genomic region-dependent: while some genomic regions that are intrinsically vulnerable to trapping, such as regions with DNA lesions that occur as part of normal cell replication, other regions where PARP1 is initially bound, as the SNAI2 promoter, respond to PARPi by PARP1 release.

To explore re-sensitization strategy, we find that SNAI2 or CHD1L depletion can re-sensitize cells with acquired resistance to Talazoparib. Further, CHD1L depletion can reduce SNAI2 expression in TalaR cells, although indirectly, but it suggests that CHD1L might act through reverting the induced SNAI2 expression. The parental cells do not rely on SNAI2 or CHD1L for survival while TalaR cells do, suggesting a growing dependence on SNAI2 and CHD1L as cells acquired drug resistance over prolonged Talazoparib treatment. As CHD1L enzymatic activity is known to be dependent on PARylation, the mechanism underlying the growing CHD1L dependency upon PARylation inhibition by PARPi treatment remains to be explored. Another plausible explanation is that CHD1L regulation of SNAI2 in TalaR cells is independent of its macro domain activity, but rather solely relies on its helicase activity and that CHD1L might substitute for PARP1 to occupy SNAI2 promoter and activate SNAI2 expression when PARP is inhibited. Thus, CHD1L activity modulation may prove efficacious in combating acquired PARP1 resistance.

## Methods

### Cell culture

Human tumor cell lines (PSN1, HCC1806, PANC1, SW1990, HS766T, PANC0504, NCIH1792, NCIH23, NCIH1651, MDAMB436) were obtained from American Type Culture Collection (ATCC) and were cultured under ATCC recommended media and condition. COV362 was obtained from Sigma-Aldrich and was maintained in DMEM media with 10% FBS. All cells were incubated at 37 °C in a humidified atmosphere with 5% CO_2_.

### Breadth of efficacy (BOE) study

Talazoparib was profiled across 334 cell lines in 9-point dose–response cell proliferation assay. Briefly, exponentially growing cells were collected, determined viability count, and resuspended in completed culture medium. Cells were seeded at the optimal density in Corning^®^ 96-well flat clear bottom black polystyrene plates TC-treated microplates (Corning) for overnight incubation. Cells were treated with threefold serial dilutions of Talazoparib starting at 3 µM for 9 dose points, or 0.1% DMSO as negative control (ZPE), and no cell wells as positive controls (HPE). Cells were cultured at 37 °C in a CO_2_ incubator for 7 days. At Day 8, cell proliferation was measured using CyQUANT Direct Cell Proliferation Assay kit (cat# C35012, Invitrogen) or Sulforhodamine B based In vitro Toxicology Assay kit (cat# TOX6-1KT, Sigma), according to the manufacturer’s instructions. Response to treatment (% of inhibition) was calculated using the formula: Y: % of inhibition = 100 − 100 × (Sample − HPE)/(ZPE − HPE). IC50 value was calculated by XLfit program.

### Computational analysis of BOE

To identify potential biomarkers of Talazoparib response in an unbiased manner, we computed Pearson correlation coefficient between the expression of individual genes and Tala response (AUC) across all cell lines from our BOE screen after excluding those with BRCA1/2 mutation or deletion. Gene expression profiles from RNA-Seq were downloaded from Cancer Cell Line Encyclopedia (CCLE) database^63^, while BRCA1/2 genetic alteration status were obtained from cBioPortal for Cancer Genomics^64^. Gene set enrichment analysis (GSEA) was then performed using a pre-ranked algorithm with genes ordered and weighted by expression-Tala response correlation and the Molecular Signature Database hallmark gene sets (version 6.2)^65^, along with default parameters weighted for enrichment statistic, meandiv for normalization mode, and timestamp as seed for 1000 rounds of permutation.

### Proteomics

For protein digestion, TMT Labeling and fractionation: cell pellets were lysed and digested as previously described^66^, except for 250 nM PARGi, final concentration, being included in the lysis buffer. Briefly, an equal volume of 8 M urea in 50 mM HEPES, pH 8.5 was added to each lysed sample, then samples were reduced with DTT and alkylated with IAA prior to methanol:chloroform precipitation. Precipitated proteins were then re-solubilized in 1 M urea in 50 mM HEPES, pH 8.5 and digested overnight with LysC at room temperature. Trypsin was added to each sample and incubated for 6 h at 37 °C to complete digestion. Digestion was quenched by addition of 10% TFA. Samples were then desalted via sep-pak, peptide concentration determined by PepQuant assay, and 50 µg aliquots of peptides made for each sample. Samples were then dried in a speed vac. A bridge channel^67^ comprised of equal portions of all samples was generated to normalize between experiments. Peptides were labeled with TMT reagents^68,69^, as previously described^70^. TMT-131C was used for the bridge channel for each plex. Samples were fractionated as previously described^71^ and then concatenated to 24 fractions, with 12 being run for mass spectrometry analysis^66^.

For LC–MS Analysis: Mass spectrometry was performed using a Thermo Orbitrap Fusion Lumos mass spectrometer. 4 µl of each sample was injected onto a 75 µm × 50 cm, 2 µm C18 column (Thermo Scientific ES803A) and separated over a 165-min gradient on an Easy nLC 1200 operated at 300 nl/min. The gradient was from 10 to 34% buffer B (80% Acetonitrile with 0.1% formic acid) for 165 min followed by a linear ramp to 100% buffer B in 5 min. After 10 min the column was returned to initial conditions and equilibrated.

The mass spectrometer was operated in data dependent mode with a five second cycle. MS1 scans were acquired in the Orbitrap with a resolution of 60,000 over a range of 500–1200 m/z. Automatic gain control (AGC) target was set to 2 × 10^5^ with a maximum inject time of 100 ms. Peptides were chosen for fragmentation if they had a charge of 2–6 and an intensity of at least 5 × 10^4^. Dynamic exclusion was enabled for 90 s. All MS2 spectra were acquired in the linear ion trap, with the quadrupole used for isolation with a window of 0.5 m/z. The AGC target for fragmentation spectra was 1 × 104 with rapid scan rate. The maximum injection time was 35 ms. Peptides were fragmented with CID at 30% normalized collision energy with an activation time of 10 ms and an activation Q of 0.25. For MS3 spectra, up to 10 ions were selected for synchronous precursor selection, and data were collected at 60,000 resolution in the Orbitrap. Ions were fragmented with HCD at an energy of 55%. MS3 AGC was set to 1 × 10^5^ with a maximum injection time of 100 ms and a first mass of 110 m/z. Data at all stages were centroided.

For data analysis: protein identification and quantification were performed with IP2GPU (IP2, Bruker Scientific LLC, Billerica, MA, http://bruker.com) using ProLuCID^72,73^, DTASelect2^74^, and Census^75,76^. Data were searched against the Human Swissprot Database (January 2018) plus sequences of known contaminants and a concatenated reverse decoy database. MS1 mass tolerance was 50 ppm (ref) and 600 ppm for MS2 data. Carbamidomethylation of Cysteine residues (+ 57.02146) and TMT modification of peptide n-termini and Lysine residues (+ 229.1629) were included as static modifications. Oxidation of Methionine (+ 15.9949) was included as a variable modification. A maximum of 2 variable modifications and two missed cleavages were allowed. Peptides had to have a minimum length of 7 amino acids to be considered. Final data were filtered to a 1% protein level false discovery rate. Pathway expression analysis was performed using the Data4Cure, Inc. Biomedical Intelligence Cloud (https://www.data4cure.com).

The mass spectrometry proteomics data have been deposited to the ProteomeXchange Consortium via the PRIDE^77^ partner repository with the dataset identifier PXD033530 and 10.6019/PXD033530.

### RNAseq

To compare transcriptome of parental, TalaR-M and TalaR-DF cells, RNA was harvested from 7 × 10^5^ cells in triplicate and stored in RNAlater RNA stabilization solution (ThermoFisher Scientific). To examine transcriptomic changes in response to Talazoparib treatment, 2.5 × 10^5^ cells in triplicate were treated with DMSO or 100 nM Talazoparib. RNA was harvested after 72 h treatment and stored in RNAlater RNA stabilization solution. RNA purification, reverse transcription, library construction and sequencing were performed at Mingma Technologies at Shanghai according to the manufacturer's instructions (Illumina). The mRNA-focused sequencing libraries from total RNA were prepared using Illumina TruSeq Stranded mRNA Library Prep kit. PolyA mRNA was purified from total RNA using oligo-dT-attached magnetic beads and then fragmented by fragmentation buffer. Taking these short fragments as templates, first strand cDNA was synthesized using reverse transcriptase and random primers. Then in the process of second strand cDNA synthesis, RNA template was removed and a replacement strand, incorporating dUTP in place of dTTP to generate ds cDNA, was synthesized. The incorporation of dUTP quenches the second strand during amplification, because the polymerase does not incorporate past this nucleotide. AMPure XP (Beckmen) beads were then used to purify the ds cDNA from the reaction mix. Next, the ds cDNA was subjected to end-repair, phosphorylation and 'A' base addition according to Illumina's library construction protocol. In the following process, Illumina sequencing adapters were added to both size of the dscDNA fragments. After PCR amplification for DNA enrichment, the AMPure XP Beads were used to clean up the target fragments of 200–300 bp. After library construction, Qubit 2.0 fluorometer dsDNA HS Assay (ThermoFisher Scientific) was used to quantify concentration of the resulting sequencing libraries, while the size distribution was analyzed using Agilent BioAnalyzer 2100 (Agilent). Sequencing was performed using an Illumina systems following Illumina-provided protocols for 2 × 150 paired-end sequencing in Mingma Technologies at Shanghai, China. Volcano plots as in Fig. 2g,h were analyzed on Data4Cure platform.

All the original RNAseq sequencing data and processed data has been deposited in GEO repository and accession number is GSE202434.

### Single cell RNA sequencing

For single-Cell RNA Sequencing Library Generation: Cells were seeded into 6-well plates at 0.3 × 10^6^ per well for overnight. Single cell suspensions were prepared according to 10 × Genomics Single Cell Sample Prep protocol. In short, cells were detached using trypsinization and washed with 10 × wash buffer (PBS plus 0.04% BSA) twice using 300 g centrifugation. After last wash, the cell numbers were counted, and viability was assessed. The final sample were at the cell density of 0.7–1.2 million/ml. About 5000 cells per sample were mixed with 10 × Single Cell 3′ v2 RT reagents and loaded onto one channel of 10X Chromium Controller (10 × Genomics, PN-1000202). After GEM generation, the RNA-seq libraries were processed following 10 × Single Cell 3′ Library Construction protocol (10X Genomics, Chromium™ Single Cell 3′ Library Construction Kit v2, PN-120237). Final libraries were sequenced by SeqMatic (44846 Osgood Rd, Fremont, CA 94539) using Illumina NavoSeq 6000 system, with 2 × 75 paired-end kits.

For Single Cell RNA sequencing Normalization and Analysis, raw sequencing reads were aligned to the human genome (hg38) using CellRanger V3.0.2. CellRanger filtered reads were imported into R V3.6.0 using Seurat V3.2.0^78^. Low quality cells were filtered from the analysis if the mitochondrial content was > 20% of reads, if the ribosomal content was > 40% of reads or if total UMI < 1500. Doublets were filtered from the analysis based on total UMI content > 30,000. Comparisons corresponding to samples which were sequenced across different batches were batch corrected with Harmony^79^ using the parameters theta = 3 and lambda = 1. To mitigate the effects of cell cycle heterogeneity in data, we followed a previously published approach by assigning each cell a score based on its expression of canonical cell phase markers and then regressing these out using Seurat. We also regressed out effects associated with the number of UMIs, mitochondrial content and ribosomal gene content using the SCTransform^80^ function in Seurat. Principal component analysis (PCA) using the top 14 principal components was performed on the top variable genes determined in Seurat. Dimensionality was reduced and data was visualized using both tSNE and UMAP representations. Phenograph clustering as a part of the Seurat package was used to discretely segregate cells into clusters. To choose an optimal resolution value, clusters were generated for resolutions spanning r = 0.1 to r = 2. The optimal resolution value was chosen ad hoc by calculating differential expression and comparing visual separation between clusters for each resolution value. After selection of a resolution value, differential gene expression was calculated using the FindAllMarkers function in Seurat using a likelihood-ratio test for single cell gene expression, selecting genes expressed in at least 20% of cells within a cluster with a fold-change > 0.2. Pathway enrichment for genes positively associated with a cluster was calculated using a hypergeometric test.

All the single cell RNAseq sequencing data and processed data has been deposited in GEO repository and accession number is GSE202435.

### Antibodies, siRNA and plasmid

The following antibodies were used in this study: Fibronectin (Abcam, ab2413), PAR (Cell signaling Technology, #83732 and Trevigen, 4336-APC-050), PARP1 (Cell Signaling Technology, #9542), TOP1 (Abcam, ab109374), Histone H3 (Cell Signaling Technology, #9715), SNAI2 (Cell Signaling Technology, #9585), Vinculin (Cell Signaling Technology, #13901), GAPDH (Cell Signaling Technology, #5174), E-Cadherin (Cell Signaling Technology, #3195), pS465/467 SMAD2 (Millipore, ab3849-I), SMAD2 (Cell Signaling Technology, #5339), SMAD4 (Abcam, ab40759), CHD1L (Cell Signaling Technology, #13460 and Abcam, ab51324), γH2AX (Bethyl Laboratories, A300-081A and Millipore, 05-636), SNAI1 (Cell Signaling Technology, #3879), TWIST1 (Cell Signaling Technology, #46702), ZEB1 (Cell Signaling Technology, #3396). ON-TARGETplus SMARTPool siRNA against human BRCA1 was purchased from Horizon Discovery and was delivered to cells by Lipofectamine RNAiMax transfection reagent (ThermoFisher). SNAI2 cDNA expressing plasmid was purchased from Origene (RC202365) and transfected into cells by using Fugene HD transfection reagent (Promega).

### Clonogenic assay

Cells were plated 2 × 10^3^ per well in 6-well plates and drug was added 24 h after plating. Colonies were allowed to form for 2–3 weeks before they were stained by 0.5% crystal violet dissolved in 50% methanol.

### PARP1-DNA trapping assay

4 × 10^6^ cells were treated with 0.001% MMS with or without 50 nM Talazoparib for 3 h before being harvested for fractionation. Subcellular Protein Fractionation Kit for Cultured Cells (ThermoFisher Scientific #78,840) was used for cellular fractionation according to manufacturer’s instructions. Nuclear soluble and chromatin bound fractions were then subjected to immunoblotting.

### Immunoblotting and immunofluorescence

For immunoblotting, cells were lysed in SDS lysis buffer (2% SDS, 10% glycerol, 0.1 M dithiothreitol and 0.2 M Tris–HCl (pH6.8)) and subjected to SDS–PAGE gel electrophoresis, and subsequently transferred to 0.2 μm PVDF membrane. Blots were incubated with the indicated primary antibodies at 4 ℃ overnight, washed by TBST and probed with corresponding horseradish peroxidase-conjugated secondary antibodies at room temperature for 2 h and subjected to ECL (Amersham). SeeBlue Plus2 pre-stained protein standard (ThermoFisher Scientific) was used as molecular weight marker. For immunofluorescence, cells were fixed with 4% paraformaldehyde, permeabilized by 0.25% Triton X-100 and blocked with 5% BSA. Cells were incubated with the indicated primary antibodies at 4 ℃ overnight. After washing four times with PBST, cells were incubated with AlexaFluo488-conjugated anti-rabbit IgG antibody (Life Technologies) at room temperature for 2 h. Nucleus was counterstained by 4,6-diamidino-2-phenylindole (DAPI). Images were taken on NIKON A1R confocal microscope.

### Annexin V flowcytometry

Annexin V flowcytometry was performed by using Annexin V Ready Flow Conjugates for Apoptosis Detection (TheromFisher, R37177) by following manufacturer’s instructions. Briefly, 1.5 × 10^6^ cells were transfected by 10 μg SNAI2 expressing plasmid DNA and were subjected to 5-day Talazoparib treatment after 48 h transfection. Cells were harvested resuspended in 500 μl 1 × binding buffer (ApoScreen Annexin V binding buffer, SouthernBiotech, 10045-01) with one drop of Annexin V Pacific Blue Ready Flow reagent and 5 μl of Propidium Iodide staining solution (BD Biosciences, 51-66211E) and were incubated at RT for 15 min. Flowcytometry analysis was conducted in BD LSRFortessa Cell Analyzer.

### CyQuant cell proliferation assay

One thousand cells per well were plated in 96-well plate 24 h before drug treatment. For BRCA1 siRNA transfected cells, cells were transfected 24 h before they were plated to 96-well plate. For SNAI2 expressing plasmid DNA transfected cells, cells were transfected 48 h before they were plated to 96-well plate. After 7-day drug treatment, CyQuant cell proliferation assay was performed by using CyQuant Direct Cell Proliferation kit (ThermoFisher, C35012) following manufacturer’s instructions. Fluorescence signal was read in Tecan plate reader.

### Quantitative RT-PCR

RNA was harvested by using PureLink RNA mini kit (ThermoFisher Scientific, 12183018A). Reverse transcription was performed on 1–5 μg of RNA by using SuperScript III First-Strand Synthesis System (ThermoFisher Scientific, 18080051). Quantitative PCR mix was prepared by iTaq Universal SYBR Green Supermix (Bio-Rad, 1725121) and was performed on QuantStudio 7 Flex System (ThermoFisher Scientific). qPCR was performed by the following conditions: 95 ℃, 30 s; 95 ℃, 3 s; 60 ℃, 3 s; 40 cycles. Primers used are the following:

**Table:** 

	Forward (5′–3′)	Reverse (5′–3′)
*SNAI2* primer set-1	GGACACACATACAGTGATTATTTCC	CTTGGACTGTAGTCTTTCCTCTTC
*SNAI2* primer set-2	GAACTGGACACACATACAGTGATTA	AAAGATGAGGAGTATCCGGAAAGAG
*TWIST1* primer set-1	GGAGACCTAGATGTCATTGTTTCC	CATAGTGATGCCTTTCCTTTCAGTG
*SNAI1* primer set-1	TCTAATCCAGAGTTTACCTTCCAGC	AAGAGACTGAAGTAGAGGAGAAGG
*SNAI1* primer set-2	TAATCCAGAGTTTACCTTCCAGCAG	AGGTATTCCTTGTTGCAGTATTTGC
*CHD1L* primer set	GCCTGGTGGGAATCCAACAATTACC	AGGGTGGGTGACATCACCACTAACG
*GAPDH* primer set	AAGGTCGGAGTCAACGGATTTG	AGAGATGATGACCCTTTTGGCTC
*TP53* primer set	GAATATTTCACCCTTCAGATCCGTG	TTCTTACATCTCCCAAACATCCCTC
*BRCA1* primer set-1	AATCTTAGAGTGTCCCATCTGTCTG	TTCTCACAGTTCCAAGGTTAGAGAG
*BRCA1* primer set-2	AATAAGGCAACTTATTGCAGTGTGG	GAGTCCGCCTATCATTACATGTTTC
*FN1* primer set-1	GAGGAAATCCAAATTGGTCACATCC	GTTCAAGCCTTCGTTGACAGAGTTG
*FN1* primer set-2	TAGGTGAGGAAATCCAAATTGGTC	TTCCAGGAACCCTGAACTGTAAGG

### ChIP qPCR

We used iDeal ChIP qPCR Kit (Diagenode, C01010180) and followed manufacturer’s instruction. Briefly, 4–7 × 10^6^ cells were used for each IP. Chromatin was sheared using Diagenode Bioruptor 300 sonication device for 10 cycles using 30 s ON and 30 s OFF setting. For each IP, 2.5 μg PARP1 antibody from ActiveMotif (61639) or Abcam (ab227244) in parallel with IgG control was used for incubation with cell lysate at 4 ℃ overnight. RNA PolII antibody was from Abcam (ab817), H3K4me3 antibody was from Abcam (ab8580). SNAI2 promoter primers used for qPCR are the following:

**Table:** 

	Forward (5′–3′)	Reverse (5′–3′)	Position on SNAI2 promoter
*SNAI2* promoter primer set-1	GCGGATCTGTGTAATGACATGCCCC	GTGCACTGCACAGATCGGCGTCAGG	2.0 kb upstream of TSS
*SNAI2* promoter primer set-3	TTCTCTGACAAGTCTTGACATCACC	GAACAAATTCACATGAAGATCACCC	1.3 kb upstream of TSS
*SNAI2* promoter primer set-7	AAAGGAGCCGGGTGACTTCAGAGGC	GTCCGGCGGGAGGACACGGCGGTCC	TSS
*SNAI2* promoter primer set-9	TGTTGAGGCTCTCCTTCCTCAATGG	TAGGTGCTCTGAAGTCAGACAGTGC	0.7 kb downstream of TSS

### Generation of stable knockdown clones by lentiviral shRNA

MISSION shRNA lentiviral transduction particles were purchased from Sigma-Aldrich. Cells were infected at MOI = 1 for 2–3 days and selected with 5 μg/ml Puromycin for 3–4 days or until all the uninfected cells were killed. Puromycin resistant cells were cultured for at least two weeks before use. shRNA clones used are the following:

**Table:** 

shRNA	Product no.	Clone no.
sh*SNAI2*-284362	SHCLNV-NM_003068	TRCN0000284362
sh*SNAI2*-271239	SHCLNV-NM_003068	TRCN0000271239
shPARP1-7928	SHCLNV-NM_001618	TRCN0000007928
shPARP1-7929	SHCLNV-NM_001618	TRCN0000007929
shPARP1-7930	SHCLNV-NM_001618	TRCN0000007930
shPARP1-7931	SHCLNV-NM_001618	TRCN0000007931
shPARP1-7932	SHCLNV-NM_001618	TRCN0000007932
shCHD1L-13469	SHCLNV-NM_004284	TRCN0000013469
shCHD1L-280395	SHCLNV-NM_004284	TRCN0000280395
shNon	SHC002V	

### CRISPR activation (CRISPRa)

Cells were first transduced by Edit-R lentiviral dCas9-VPR particles (Horizon Discovery) with human CMV promoter (VCAS11918) at MOI = 1 for 2–3 days and selected with 5 μg/ml Blasticidin for 3–4 days or until all the uninfected cells were killed. The Blasticidin resistant cells were then transduced with Edit-R CRISPRa human *SNAI2* lentiviral sgRNA (Horizon Discovery, VSGH11888-247237086 and VSGH11888-247237089) at MOI = 1 for 2–3 days and selected with 5 μg/ml Puromycin for 3–4 days or until all the uninfected cells were killed.

### Inducible shRNA

Lentiviral doxycycline inducible shRNA was designed and generated at Transomic Technologies, Inc by using the shERWOOD-UltramiR algorithm and pZIP-TRE3G as backbone vector. Inserted shRNA sequences are listed as below (target sequences are underlined). Puromycin was used as antibiotic selection marker. Cells were infected at MOI = 1 for 3 days and selected with 5 μg/ml Puromycin for 3–4 days. For IncuCyte scanning, stable cells were plated at 2000 cells per well in 48-well plate and doxycycline was added at 2.5 μM for induction 24 h after plating.

**Table:** 

shNon	TGCTGTTGACAGTGAGCGAAGGCAGAAGTATGCAAAGCATT AGTGAAGCCACAGATGTAATGCTTTGCATACTTCTGCCTGT GCCTACTGCCTCGGA
shCHD1L	TGCTGTTGACAGTGAGCGAGAAGAGGTGGGAGATTTTATAT AGTGAAGCCACAGATGTATATAAAATCTCCCACCTCTTCCT GCCTACTGCCTCGGA

### ATACseq

Permeabilized nuclei were obtained by resuspending cells in 250 µL Nuclear Permeabilization Buffer [(10 mM Tris–HCl (pH 7.4) (BP-152, Thermo Fisher Scientific), 10 mM NaCl (S271-3, Thermo Fisher Scientific), 3 mM MgCl2 (194698, MP Biomedicals), 0.1% Tween-20 (P7949, Sigma), 0.1% IGEPAL-CA630 (I8896, Sigma), 0.01% digitonin (G9441, Promega) in Molecular biology water (46000-CM, Corning)], and incubating for 5 min on a rotator at 4 °C. Nuclei were then pelleted by centrifugation for 5 min at 500 × *g* at 4 °C. The pellet was resuspended in 25 µL ice-cold Tagmentation Buffer [33 mM Tris–acetate (pH = 7.8) (BP-152, Thermo Fisher Scientific), 66 mM K-acetate (P5708, Sigma), 11 mM Mg-acetate (M2545, Sigma), 16% DMF (DX1730, EMD Millipore) in Molecular biology water (46000-CM, Corning)]. An aliquot was then taken and counted by hemocytometer to determine nuclei concentration. Approximately 50,000 nuclei were resuspended in 10 µL ice-cold Tagmentation Buffer, and incubated with 1 µL Tagmentation enzyme (FC-121-1030; Illumina) at 37 °C for 30 min with shaking 500 rpm. The tagmentated DNA was purified using MinElute PCR purification kit (28004, Qiagen). The libraries were amplified using NEBNext High-Fidelity 2X PCR Master Mix (M0541, NEB) with primer extension at 72 °C for 5 min, denaturation at 98 °C for 30 s, followed by 8 cycles of denaturation at 98 °C for 10 s, annealing at 63 °C for 30 s and extension at 72 °C for 60 s. Amplified libraries were then purified using MinElute PCR purification kit (28004, Qiagen), and two size selection steps were performed using SPRIselect bead (B23317, Beckman Coulter) at 0.55X and 1.5X bead-to-sample volume rations, respectively. All libraries were sequenced on an Illumina NovaSeq 6000 in paired-end mode.

All the original ATACseq sequencing data and processed data has been deposited in GEO repository and accession number is GSE202437.

### IncuCyte analysis

Cells were plated at a density of 2000 cells per well in 48-well plate with 1 ml of media. Scanning was initiated 24 h after plating. Cell growth was monitored by 10× objective and analyzed on IncuCyte ZOOM or IncuCyte S3 (Essen BioScience).

### Gene knockout by CRISPR/Cas9 ribonucleoprotein (RNP) electroporation

Custom designed synthetic single gRNA (sgRNA, 100 mer) and Alt-R S.p. Cas9 nuclease (Cat#0.1081059) were purchased from Integrated DNA Technologies (IDT). sgRNA sequences are listed below. sgRNA was diluted in IDT duplex buffer at stock concentration of 200 μM. Electroporation was performed by using Neon transfection system (ThermoFisher Scientific). RNP (sgRNA and Cas9) and cells were mixed as follows: sgRNA (2.5 μM final concentration), Cas9 (2.5 μM final concentration) and 1 × 10^6^ cells were resuspended in 100 μl total volume in Buffer R which was subjected to the pulse. Specifically, RNP mix was prepared and incubated at room temperature for at least 10 min before mixing with cells. Cells were trypsinized, rinsed with PBS and resuspended in Buffer R. Electroporation pulse was delivered by Program #5 (Pulse Voltage: 1700 V, Pulse Width: 20 ms, Pulse Number: 1). Cells were recovered in 6-well plate for at least 3 days before used for downstream analysis. For determine *SNAI2* knockout efficiency as in Fig. 3o, we quantified indel percentage as follows: DNA was extracted 3–5 days post electroporation by using QuickExtract DNA Extraction Solution (Lucigen). We used specific primers (forward: 5′-GTGAGTTCTAATGTGTCCTTGAAGC-3′, reverse: 5′-GAAGAGGAAAGACTACAGTCCAAGC-3′) flanking sgRNA regions and performed PCR. PCR product was sequenced at Genewiz and indel percentage and knockout score was analyzed by ICE analysis (Synthego).

**Table:** 

sgRNAs	Sequences (5′–3′)
OR2B2 sgRNA	GTCTGCTGAAGGTCAACGAA
SNAI2 sgRNA	GACAAGGAATATGTGAGCCT
CHD1L sgRNA-1	TTCGTGGCATGTCCAATACG
CHD1L sgRNA-2	CATCACCACTAACGTACTTG
CHD1L sgRNA-3	ACACCTGGCAGGTCTTCCCC

### Statistics

Statistics was performed by two-tailed t-test. All error bars represent standard deviation. *P* < 0.05 was considered statistically significant. Asterisks represent *P < 0.05, **P < 0.01, ****P < 0.0001. No statistical methods or criteria were used to estimate sample size or to include or exclude samples.

## Supplementary Information

Supplementary Legends.

Supplementary Figures.

Supplementary Table 1.

Supplementary Information.

## Data Availability

The genomic sequencing datasets including RNAseq, single cell RNAseq and ATACseq generated and analyzed during the current study are available in the GEO repository. The accession number is GSE202438. Accession link: https://www.ncbi.nlm.nih.gov/geo/query/acc.cgi?acc=GSE202438, https://www.ncbi.nlm.nih.gov/geo/query/acc.cgi?acc=GSE202434, https://www.ncbi.nlm.nih.gov/geo/query/acc.cgi?acc=GSE202435, https://www.ncbi.nlm.nih.gov/geo/query/acc.cgi?acc=GSE202437. The proteomics datasets generated and analyzed during the current study are available in the PRIDE repository. The accession number is PXD033530. Accession link: https://www.ebi.ac.uk/pride/archive/projects/PXD033530.
