# Supplementary material for: PARP1-SNAI2 transcription axis drives resistance to PARP inhibitor, Talazoparib
Source: Sci Rep. 2022 Jul 21;12:12501. doi: 10.1038/s41598-022-16623-3 (PMC9304387; doi:10.1038/s41598-022-16623-3)
Supplement: Supplementary file 1 — Supplementary Legends. [file 41598_2022_16623_MOESM1_ESM.doc]

**Supplementary Figure S1. Detailed information on Talazoparib BOE analysis. (a)** Pie chart showing number of cell lines in each tumor lineage included in Talazoparib BOE. **(b)** Violin plot of IC50 across tumor lineages. **(c)** Scattered plot showing positive correlation between PARP1 mRNA level with increased Talazoparib sensitivity as in BOE. AUC, area under curve. **(d)** Cell lines sorted by Talazoparib response, with genetic alteration status in 14 non-BRCA DDR genes shown in the heatmap below. The last row (DDR) represents their union where “Y” indicates any of the 14 genes harbors alteration. LOF refers to loss-of-function alterations (deletions, frameshifts and truncations). The statistical significance of difference in Talazoparib response between altered and wild type groups are shown on the right. It was determined using Wilcoxon rank sum test (also known as Mann-Whitney test). Those with nominal P-value <0.05 are marked by asterisks. **(e, f)** Talazoparib response (AUC) with cell lines divided by genetic alteration in ATR and NBN. Statistical significance of their difference was determined using Wilcoxon rank sum test.

**Supplementary Figure S2. Detailed characterization of cell lines with acquired resistance to Talazoparib. (a)** Clonogenic assay showing cell lines with acquired resistance to Talazoparib. **(b)** IC_50_ chart of cross-resistance to five different PARPis in PSN1 TalaR cells. **(c)** Immunoblot showing PAR level of parental and TalaR cells in PSN1 and HCC1806 cells. **(d)** Immunoblot showing PARP1-DNA trapping level in PSN1 and PANC1 parental and TalaR cells. **(e)** Immunofluorescence showing DNA damage as indicated by increased γH2AX foci in PSN1 parental and TalaR cells. Scale bar = 20 μm. **(f)** Quantification of average γH2AX foci number per nuclear as shown in **(e)**. **(g)** Normalized cell viability as measured by 7-day CyQuant cell proliferation assay of PSN1 parental and TalaR cells treated with cisplatin. (h) Immunoblot showing PAR level in HCC1806 parental, TalaR-DF and TalaR-M cells. **(i)** Heatmap showing hallmark pathways based on total proteome by mass spectrometry (MS) of PANC1 parental and TalaR cells, n = 5 for each group as experimental replicates. **(j, k)** Heatmap showing hallmark pathways based on RNAseq of PANC1 and HCC1806 parental and TalaR cells, n = 3 for each group as experimental replicates.

**Supplementary Figure S3. Talazoparib treatment or PARP1 KD induces EMT signature and SNAI2. (a, b, c)** Heatmap showing hallmark pathways based on RNAseq of HS766T, PANC1 and SW1990 cells treated with Talazoparib (50 nM, 72 hr). n = 3 for each group as experimental replicates. **(d)** qPCR showing mRNA level of *TWIST1* and *SNAI1* was not increased under Talazoparib treatment in PSN1 cells. **(e)** Normalized cell viability as measured by 7-day CyQuant cell proliferation assay of PSN1 cells transfected by BRCA1 siRNA followed by Talazoparib treatment. **(f)** qPCR showing BRCA1 KD level in PSN1 cells after siRNA transfection. **(g, h)** qPCR showing dose-dependent induction of *SNAI2* mRNA by Talazoparib in two *BRCA1*-mutated cell lines, COV362 (ovarian) and MDAMB436 (breast).

**Supplementary Figure S4. SNAI2 modulates cellular sensitivity to Talazoparib. (a)** Normalized cell viability as measured by 7-day CyQuant cell proliferation assay of PSN1 cells transfected with empty vector (EV) or SNAI2 expressing vector (SNAI2) followed by Talazoparib treatment. **(b)** qPCR showing dose-dependent induction of *FN1* mRNA by Talazoparib in PSN1 cells. **(c)** qPCR showing *FN1* mRNA level in PSN1 cells transfected by empty vector (EV) or SNAI2 expressing vector (SNAI2). **(d)** Percentage of apoptotic cells as measured by Annexin V flowcytometry in PSN1 or MDAMB436 cells transfected by empty vector (EV) or SNAI2 expressing vector (SNAI2) followed by Talazoparib treatment. **(e)** Clonogenic assay showing sensitivity to Talazoparib after PSN1 cells were transduced with lentiviral shRNA against *SNAI2.* Two shRNA clones were used: 271239 and 284362*.* **(f)** qPCR showing Talazoparib-induced *SNAI2* was abolished in cells transduced with lentiviral shRNA against *SNAI2*. These cells were used for clonogenic assay as in **(e)**.

**Supplementary Figure S5. Talazoparib induced SNAI2 expression is independent of TGFβ and p53 activation. (a, b)** qPCR showing mRNA level of *TWIST1* and *SNAI1* was not increased in PSN1 and PANC1 cell lines with PARP1 stable knockdown. **(c)** Immunoblot showing phosphorylated level of SMAD2, total level of SMAD2 and SMAD4 under Talazoparib or TGFβ treatment in PSN1 and PANC1 cells. **(d)** qPCR showing SNAI2 mRNA levels under different treatment regimens in PSN1 cells. LY3200882 is a TGFβR1 inhibitor. **(e)** qPCR showing *TP53* mRNA level in PSN1 cells with siRNA transient knockdown. **(f)** qPCR showing Talazoparib-induced SNAI2 expression in PSN1 cells with *TP53* siRNA and nonsense control siRNA.
