# Supplementary material for: PARP1-SNAI2 transcription axis drives resistance to PARP inhibitor, Talazoparib
Source: Sci Rep. 2022 Jul 21;12:12501. doi: 10.1038/s41598-022-16623-3 (PMC9304387; doi:10.1038/s41598-022-16623-3)
Supplement: Supplementary file 2 — Supplementary Figures. [file 41598_2022_16623_MOESM2_ESM.pdf]

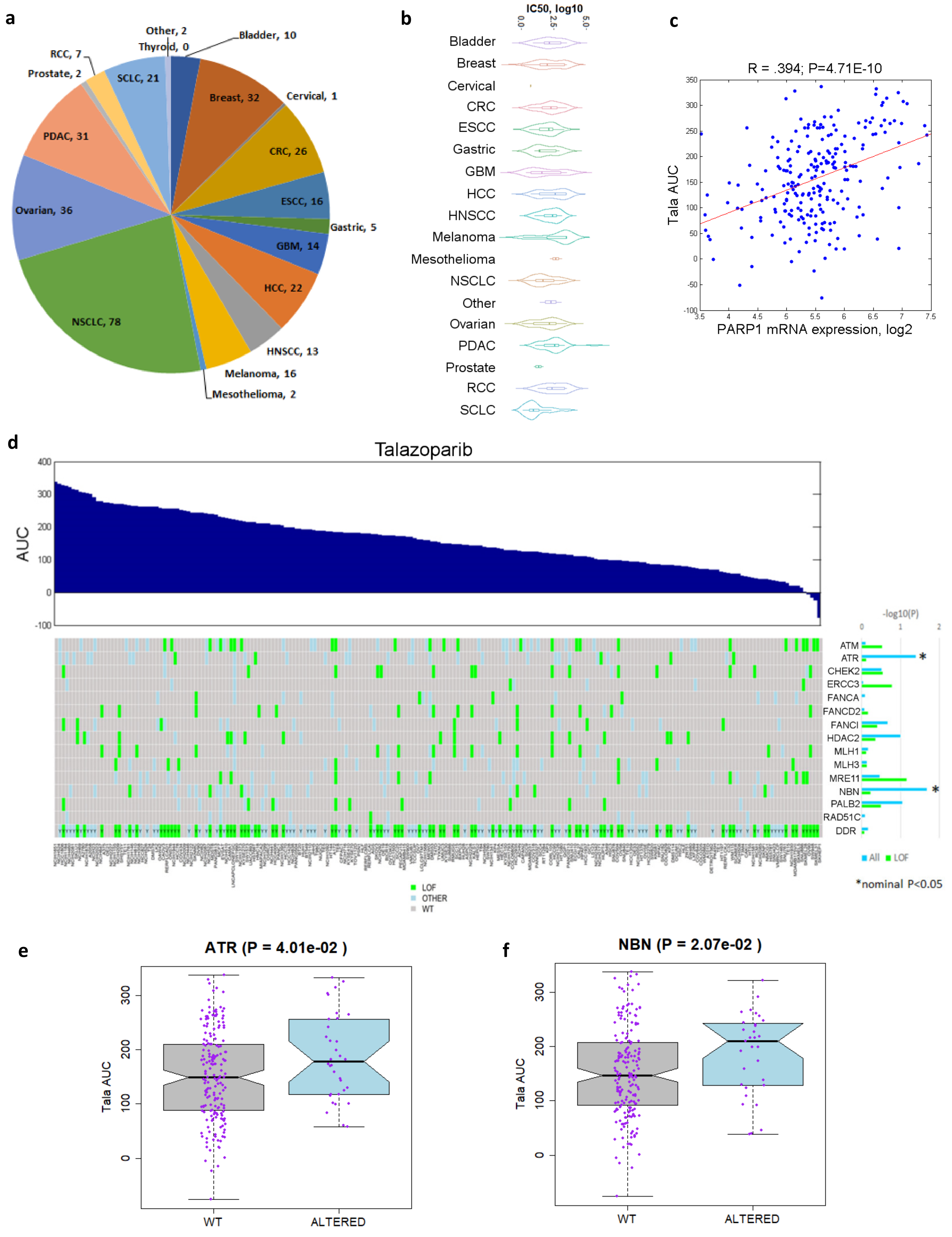

Supplementary Figure S1: Detailed information on Talazoparib BOE analysis

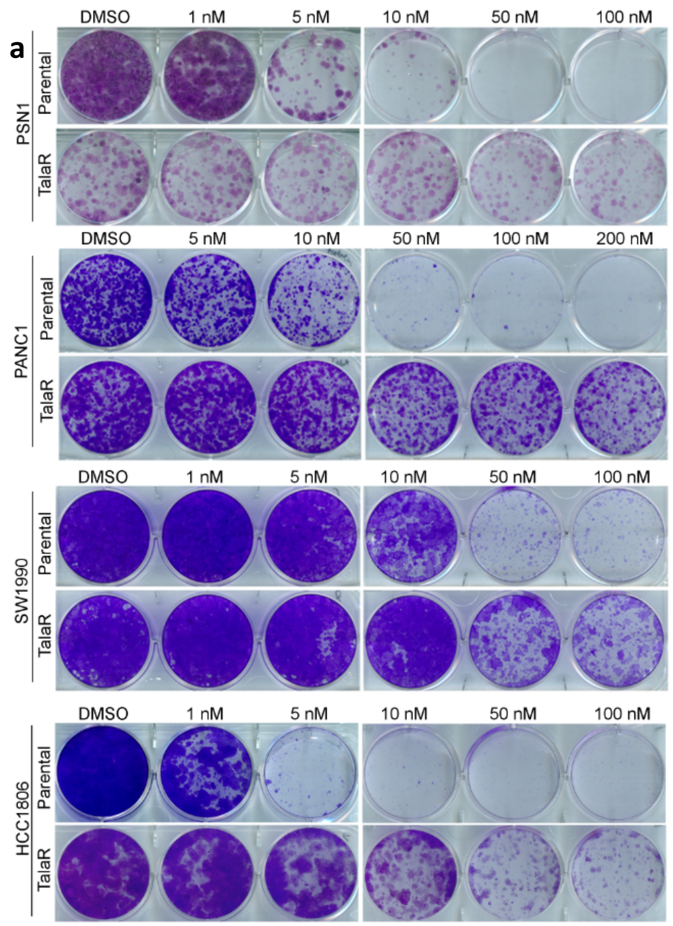

b

PARPi	PSN1 parental IC50 (nM)	PSN1 TalaR IC50 (nM)	Fold increase (TalaR vs parental)
Talazoparib	7	268	38x
Olaparib	214	2568	12x
Niraparib	148	1095	7x
Veliparib	3029	26156	9x
Rucaparib	188	1071	6x

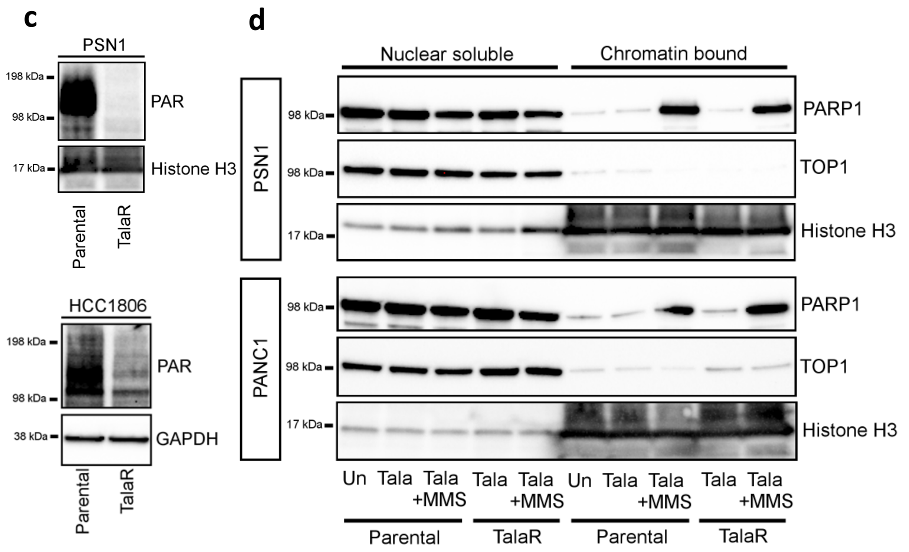

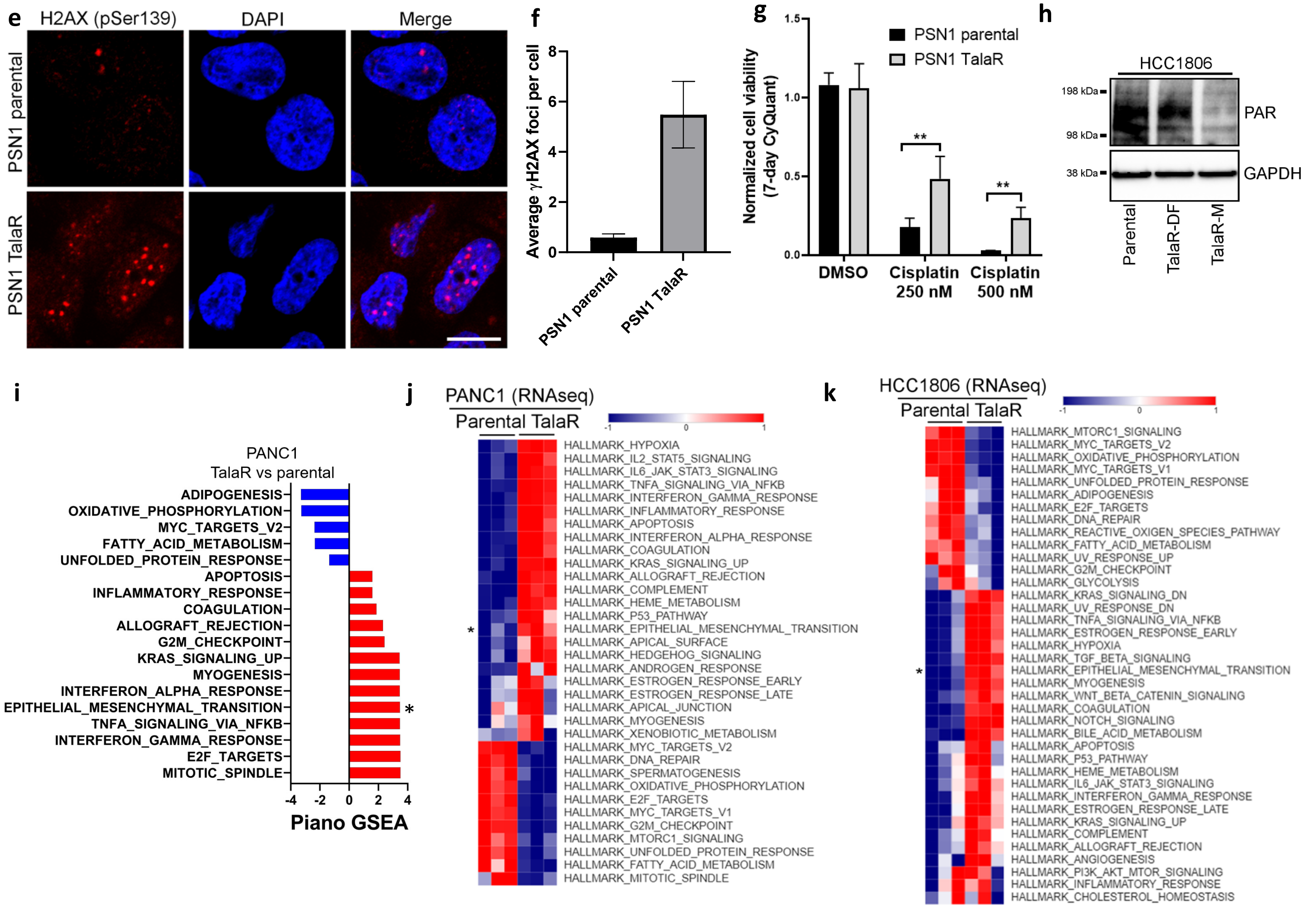

Supplementary Figure S2: Detailed characterization of cell lines with acquired resistance to Talazoparib

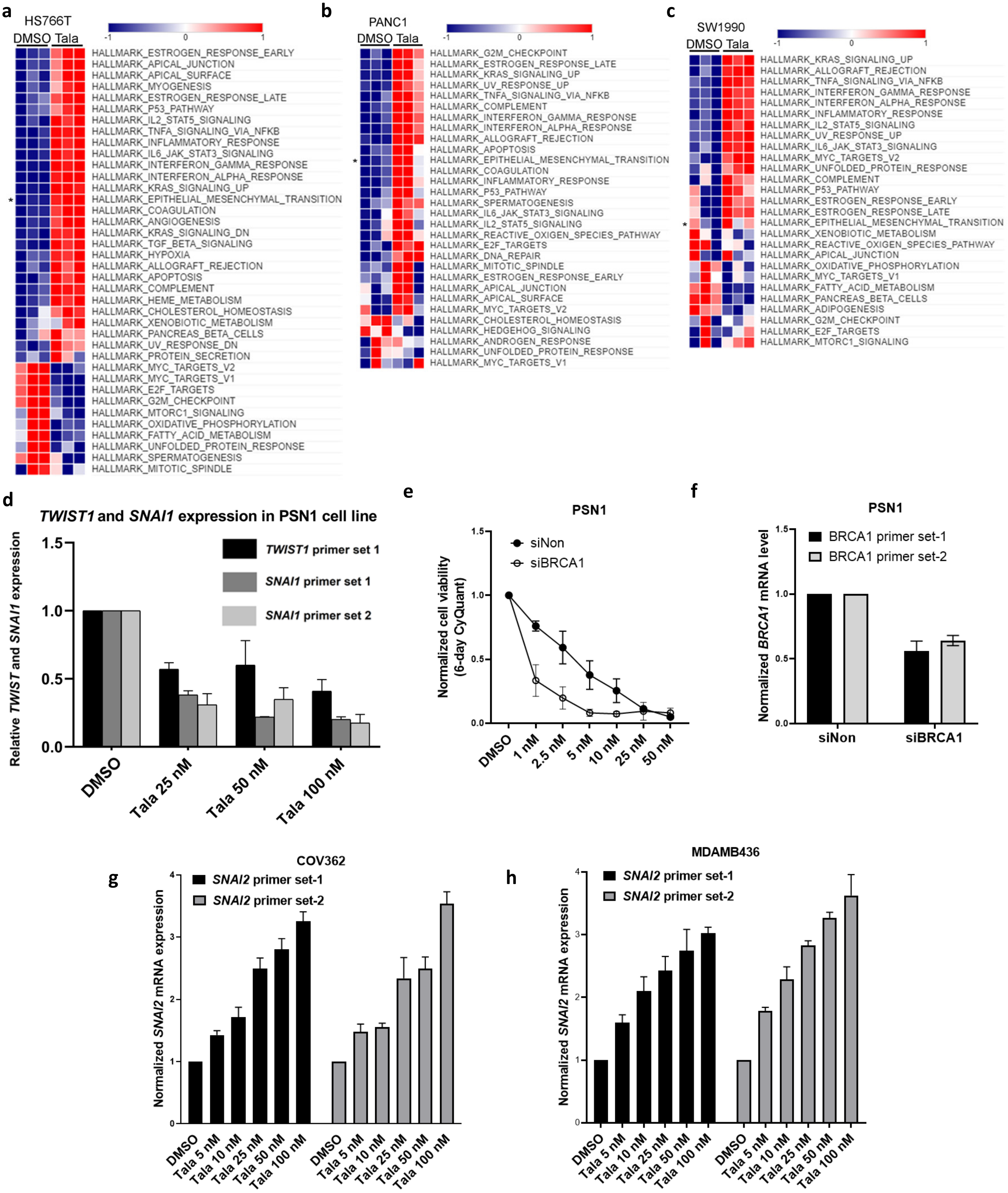

Supplementary figure S3. Talazoparib treatment or PARP1 KD induces EMT signature and SNAI2

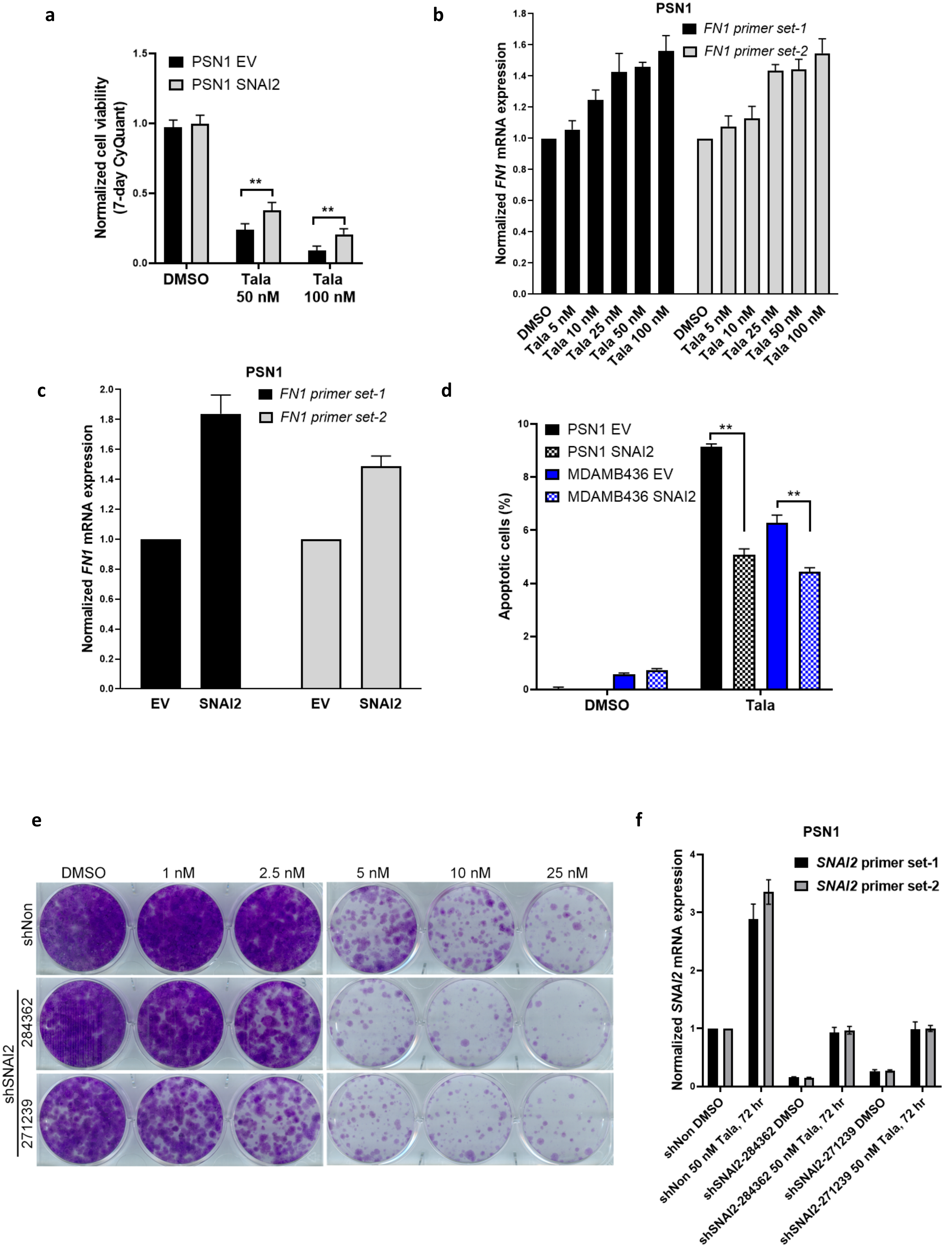

Supplementary figure S4. SNAI2 modulates cellular sensitivity to Talazoparib

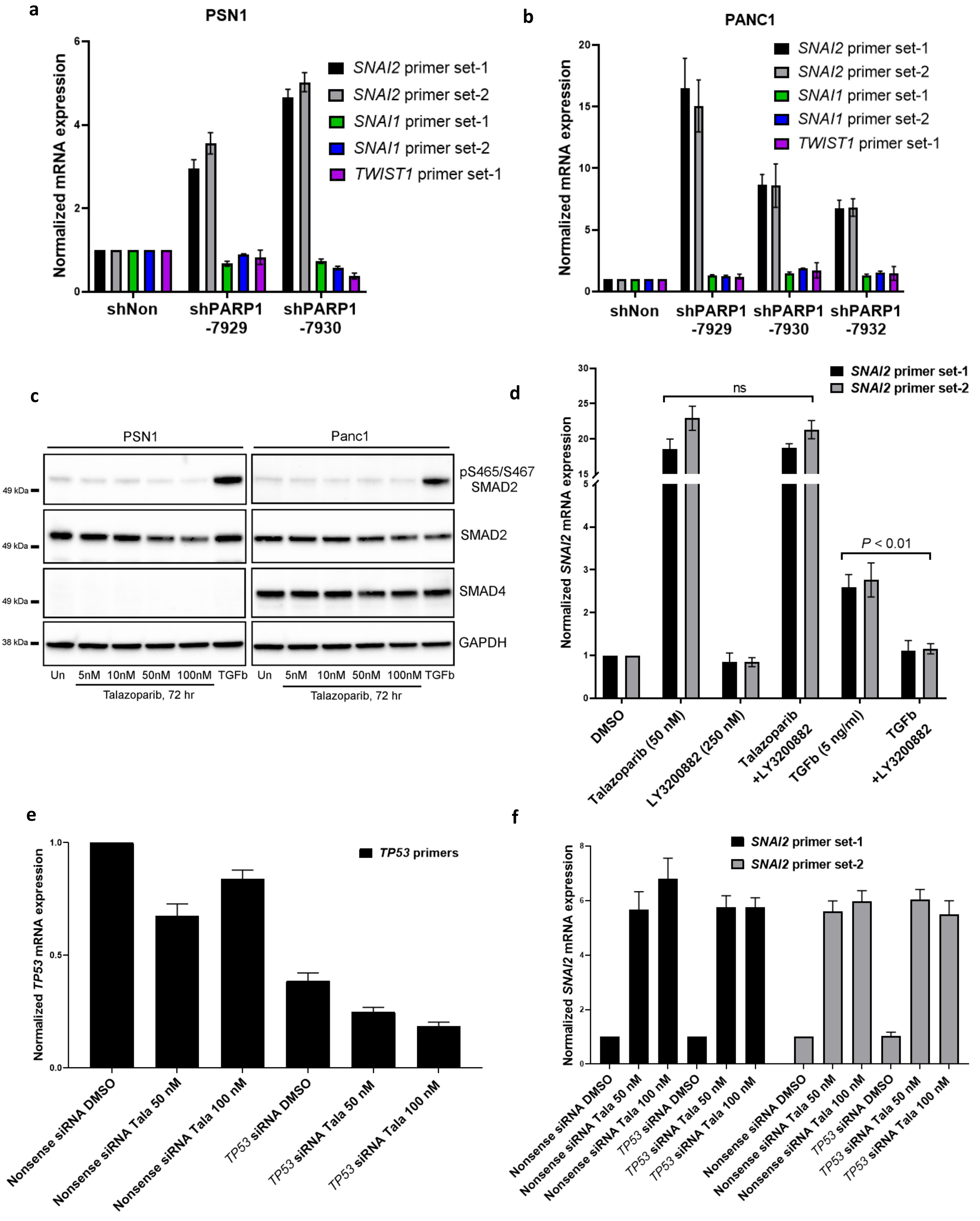

Supplementary figure S5. Talazoparib-induced SNAI2 expression is independent of TGFβ and p53 activation
